# Exploring the Association Between Per‐ and Polyfluoroalkyl Substances Exposure and the Risk of Stroke: A Systematic Investigation Using NHANES Data Analysis, Network Toxicology and Molecular Docking Approaches

**DOI:** 10.1002/brb3.71014

**Published:** 2025-10-24

**Authors:** Yanjie Jiang, Ling Li, Shipeng Zhang, Man Lu, Xingyi He, Rui Fu, Mingjie Tang, Yinghong Li, Qinwei Fu, Zhihui Jin, Yupeng Weng, Wenshan Li, Xiaoyu Zhu, Enjie Tang, Hanyu Wang, Yan Lu

**Affiliations:** ^1^ Nanjing Hospital of Chinese Medicine Affiliated to Nanjing University of Chinese Medicine Nanjing China; ^2^ Department of Neurology 925 Hospital of PLA Joint Logistics Support Force Guiyang China; ^3^ Hospital of Chengdu University of Traditional Chinese Medicine Chengdu University of Traditional Chinese Medicine Chengdu China; ^4^ No. 1 Clinical Medical College Nanjing University of Chinese Medicine Nanjing China; ^5^ The First Clinical Medical College Guangzhou University of Chinese Medicine Guangzhou China; ^6^ Department of Health Research Methods, Evidence, and Impact (HEI) McMaster University Hamilton Canada; ^7^ Department of Rehabilitation Sciences, Faculty of Health and Social Sciences The Hong Kong Polytechnic University Hong Kong SAR China

**Keywords:** molecular docking, NHANES, network toxicity, per‐ and polyfluoroalkyl substances, stroke

## Abstract

**Background:**

Epidemiologic evidence regarding the association between per‐ and polyfluoroalkyl substances (PFAS) exposure and stroke risk remains limited and inconclusive. Consequently, the current study sought to further examine this association and clarify the underlying molecular mechanisms.

**Materials and Methods:**

This cohort study analyzed data from 8081 participants of the 2003–2012 National Health and Nutrition Examination Survey (NHANES), employing multistage weighted logistic regression, weighted quantile sum (WQS) modeling, and partial least squares discriminant analysis (PLS‐DA) to systematically evaluate the association between per‐ and polyfluoroalkyl substances (PFAS) exposure and stroke. Restricted cubic spline analysis was subsequently used to examine the nonlinear dose‐response relationships. To investigate the underlying mechanisms, we integrated data from six databases (e.g., ChEMBL and GeneCards) to identify common molecular targets of PFAS and stroke. A protein‐protein interaction (PPI) network was then constructed to identify core genes, while the binding interactions between PFAS and key targets were evaluated through molecular docking and dynamics simulations. Finally, functional enrichment analysis was performed on these core genes using the Gene Ontology (GO) and Kyoto Encyclopedia of Genes and Genomes (KEGG) databases.

**Results:**

After adjusting for potential confounders, six individual PFAS compounds, including perfluorooctane sulfonic acid (PFOS) (Odds Ratio [OR] = 1.59, 95% CI: 1.09–2.31), exhibited a significant positive association with the risk of stroke. The WQS model revealed a significant positive association for the PFAS mixture (OR = 1.027, 95% CI: 1.017–1.036), with PFOS contributing the highest weight (0.379). These findings were corroborated by the PLS‐DA model, and the association remained significant in all subgroup analyses. The network toxicology analysis identified 183 common targets between PFOS and stroke, while the subsequent PPI network analysis identified six core genes, including AKT1 and HSP90AA1. GO and KEGG enrichment analyses demonstrated that these targets were markedly enriched in pathways associated with lipid and atherosclerosis metabolism, in addition to the PI3K‐Akt and MAPK signaling pathways. Furthermore, molecular docking and molecular dynamics simulations supported potential interactions between PFOS and core targets such as AKT1. This suggests that PFOS may contribute to stroke pathogenesis by disrupting pathways involved in inflammatory regulation and apoptosis.

**Conclusions:**

This study identified a positive association between PFOS exposure and stroke risk, suggesting that the PI3K/AKT signaling pathway, along with its key effector molecule AKT1, may play a crucial role in mediating PFOS‐induced stroke, thereby offering a theoretical foundation for the prevention and management of PFOS‐associated stroke.

## Graphical abstract

This comprehensive analysis, integrating NHANES data with computational modeling, reveals a significant association between PFOS exposure and an increased risk of stroke. Among a cohort of 8,081 U.S. adults, PFOS exhibited the strongest association in both multivariate and mixed‐effects models. Network toxicology analysis identified 183 putative targets common to both PFOS and stroke, with the core genes (AKT1, HSP90AA1, EGFR, SRC, ESR1, TNF) being prominently enriched in pathways such as the PI3K‐Akt and MAPK signaling pathways. Furthermore, molecular docking and kinetic simulations revealed stable binding between PFOS and AKT1, suggesting that PFOS may disrupt neurovascular homeostasis through inflammatory and apoptotic signaling pathways.

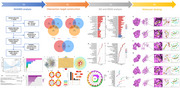

**Abbreviations**
BBB: blood‐brain barrier
BMI: Body Mass Index
BP: Biological Process
CC: Cellular Components
CHD: Coronary heart disease
CTD: Comparative Toxicogenomics Database
CVD: Cardiovascular diseases
GBD: Global burden of disease
GO: Gene Ontology
KEGG: Kyoto Encyclopedia of Genes and Genomes
MF: Molecular Functions
N‐ETFOSAA: 2‐(N‐Ethyl‐perfluorooctane sulfonamido) acetic acid
NHANES: National Health and Nutrition Examination Surveys
N‐MEFOSAA: 2‐(N‐Methyl‐perfluorooctane sulfonamido) acetic acid
OR: Odds Ratio
PA: Physical activity
PFAS: per‐ and polyfluoroalkyl substances
PFBS: Perfluorobutane sulfonic acid
PFDA: Perfluorodecanoic acid
PFDO: Perflurododecanoic acid
PFHPA: Perfluoroheptanoic acid
PFHXS: Perfluorohexane sulfonic acid
PFNA: Perfluorononanoic acid
PFOA: Perfluorooctanoic acid
PFOS: Perfluorooctane sulfonic acid
PFOSA: Perfluorooctane sulfonamide
PFUA: Perfluoroundecanoic acid
PIR: Poverty Income Ratio
PLS‐DA: Partial Least Squares Discriminant Analysis
RCS: Restricted cubic splines
WQS: Weighted quantile sum regression

## 1 Introduction

Per‐ and polyfluoroalkyl substances (PFAS) are a class of synthetic organofluorine compounds characterized by exceptional chemical and thermal stability. This exceptional stability has led to their extensive use in various industrial and consumer products, such as food packaging, textiles, cleaning agents, and firefighting foams (Panieri et al. 2022, Figuière et al. 2025). Consequently, their persistence and widespread use have resulted in extensive environmental contamination. Global annual production of fluoropolymers exceeds 230,000 tons, with estimated total PFAS emissions surpassing 46,000 tons (Johansson et al. 2019). In response to these concerns, legacy compounds such as perfluorooctanoic acid (PFOA) and perfluorooctane sulfonic acid (PFOS) were listed for elimination under the Stockholm Convention on Persistent Organic Pollutants in 2019 (United Nations Environment Programme. 2023). Their long biological half‐lives contribute to their classification as “persistent chemicals”, resulting in bioaccumulation in living organisms and persistence in the environment (Liang et al. 2022). This bioaccumulation is evident from their detection in diverse human tissues, including blood, urine, placenta, and bone (Domingo 2025). The combination of historical emissions, environmental persistence, and bioaccumulation has raised significant concerns regarding the ongoing health risks of PFAS exposure (Fair et al. 2019). Underscoring this concern, data from the National Health and Nutrition Examination Survey (NHANES) reveal that serum PFAS prevalence in the United States population has remained above 78% between 1999 and 2020 (Botelho et al. 2025). Indeed, a growing body of evidence from both epidemiological studies and laboratory trials links PFAS exposure to a spectrum of adverse health outcomes, including endocrine disruption (Jane et al. 2022), reproductive impairments (Ding et al. 2020), and cardiovascular diseases (Wen et al. 2023), among other risks.

Stroke is the third‐leading cause of mortality worldwide and the primary driver of long‐term disability, affecting over ten million individuals annually (GBD 2021 Causes of Death Collaborators 2024). Furthermore, projections from the Global Burden of Disease (GBD) study estimate that stroke‐related mortality, disability‐adjusted life years, and economic costs are poised to nearly double between 2020 and 2050, with over 80% of this burden disproportionately affecting low‐ and middle‐income countries (Feigin et al. 2023). The GBD 2021 systematic analysis identified 23 modifiable risk factors significantly associated with stroke incidence (GBD 2021 Stroke Risk Factor Collaborators 2024). Importantly, in addition to traditional risk factors, the role of environmental toxins (e.g., PM2.5, heavy metals) in the pathophysiology of stroke is increasingly being recognized (Chowdhury et al. 2018). However, the association between PFAS exposure and stroke specifically remains controversial and understudied, with existing research often limited by methodological constraints (Huang et al. 2018, Meneguzzi et al. 2021). In a Swedish dual‐cohort analysis, researchers observed no statistically significant associations between serum concentrations of PFHxS, PFOA, or PFOS and the onset of cardiovascular diseases (CVD), including myocardial infarction and ischemic stroke (Dunder et al. 2023). Moreover, a pooled analysis of five distinct investigations demonstrated that participants with elevated PFAS concentrations exhibited a reduced CVD incidence (hazard ratio = 0.80, 95% CI: 0.66–0.94), indicating a possible inverse relationship (Dunder et al. 2023). A subsequent systematic review targeting stroke risk arrived at comparable results (Chang et al. 2024). By contrast, the inaugural Italian cohort tracked over thirty years documented a significant rise in cardiovascular mortality linked to PFAS exposure (Biggeri et al. 2024). Additional research has identified a positive association between higher circulating PFOA levels and greater CVD risk (Mao et al. 2024). Given these conflicting epidemiological findings and a general lack of mechanistic inquiry, the precise role of PFAS in the etiology of stroke remains unresolved. This knowledge gap represents a critical barrier to developing targeted public health strategies for stroke prevention.

While some epidemiological studies and meta‐analyses have reported no significant association between PFAS exposure and stroke risk and have even suggested a potential paradoxical protective effect, these findings stand in stark contrast to the well‐established endocrine‐disrupting and pro‐inflammatory properties of PFAS (Wen et al. 2023, Zhang et al. 2023, Zhang et al. 2024). The discrepancy in these findings may be attributable to several factors, including inherent confounding biases in epidemiological research (e.g., the healthy worker effect), challenges in assessing the effects of individual PFAS components due to co‐exposure to other environmental toxins, heterogeneity in study populations and endpoint definitions (e.g., combining stroke with myocardial infarction), and potential non‐linear dose‐response relationships. Such methodological limitations hinder the ability of traditional linear models to accurately capture the true exposure‐effect relationship, resulting in divergent conclusions. To systematically address these challenges, this study utilized nationally representative data from the National Health and Nutrition Examination Survey (NHANES, 2003–2012), featuring PFAS concentrations that are representative of contemporary global general population levels (Botelho et al. 2025). We employed a multi‐faceted modeling approach, integrating weighted quantile sum (WQS) regression, multivariate logistic regression, and partial least squares discriminant analysis (PLS‐DA), to comprehensively evaluate the effects of PFAS mixtures and identify key contributing components. Furthermore, we utilized multiscale computational methods—including network toxicology, molecular docking, and molecular dynamics simulations—to investigate the biological mechanisms, potential association pathways, and molecular basis of action linking PFOS to stroke risk. A detailed research workflow is shown in the **GRAPHICAL ABSTRACT**. This comprehensive approach provides a novel evidence chain and mechanistic foundation, aiming to clarify the existing controversies surrounding PFAS exposure and stroke risk.

## 2 Materials and Methods

### 2.1 Study Design

This study employed a comprehensive two‐stage analytical framework to systematically investigate the association between PFAS exposure and stroke risk, along with the underlying molecular mechanisms (**GRAPHICAL ABSTRACT**). In the first stage, we conducted an epidemiological analysis using data from the NHANES 2003–2012 cohort. Individual PFAS congeners significantly associated with stroke were identified through multistage weighted regression modeling, while their combined effects were evaluated using mixed exposure models. In the second stage, a mechanistic investigation was performed. This involved in silico toxicity prediction of the key PFAS compounds, followed by a network toxicology approach to construct a multilevel interaction network integrating chemical structures, biological targets, and signaling pathways. To corroborate key interactions identified from the network analysis, molecular docking and molecular dynamics (MD) simulations were performed to uncover potential molecular mechanisms underlying PFAS‐induced stroke. All data were obtained from publicly available databases, including NHANES, PubChem, ChEMBL, and the Comparative Toxicogenomics Database (CTD), as detailed in Supplementary **eTable 1**.

### 2.2 Study Population and Data Collection

NHANES is a cross‐sectional national study designed to assess the health and nutritional status of non‐institutionalized adults and children in the United States, conducted by the Centers for Disease Control and Prevention (CDC) (Botelho et al. 2025). Researchers administered questionnaires during home visits and collected biospecimens at mobile examination centers (MEC) (Centers for Disease Control and Prevention [CDC] National Center for Health Statistics [NCHS]. 2016).

This study screened data from participants enrolled between 2003 and 2012, with a total of 50,912 participants included. Exclusion criteria were applied sequentially: **(1)** age < 20 years (*N* = 23,179); **(2)** missing data on stroke history (*N* = 88); and (3) incomplete PFAS exposure data (*N* = 19,564). The final analysis included 8081 participants, representing a weighted population of 21.32 million people in the United States after adjusting for the NHANES complex sampling design.

### 2.3 Measurement of PFAS Compounds

Serum concentrations of PFAS were quantified by the United States National Center for Environmental Health across five NHANES cycles (2003–2012) using solid‐phase extraction coupled with high‐performance liquid chromatography–tandem mass spectrometry (HPLC‐MS/MS). Quantification was performed using negative ion electrospray ionization, with limits of detection (LODs) typically in the low nanogram‐per‐milliliter (ng/mL) range (Cui et al. 2025). These measurements were conducted on a random, representative subsample comprising approximately one‐third of the total NHANES population for each cycle. All laboratory procedures adhered to the quality assurance and quality control (QA/QC) protocols mandated by the Clinical Laboratory Improvement Amendments of 1988 (CLIA) (Kuklenyik et al. 2005). Of the twelve PFAS congeners measured during this period, ten with a detection rate exceeding 50% were included in the present analysis: perfluorooctanoic acid (PFOA), perfluorooctane sulfonic acid (PFOS), perfluorohexane sulfonic acid (PFHxS), 2‐(N‐ethyl‐perfluorooctane sulfonamido) acetic acid (EtFOSAA), 2‐(N‐methyl‐perfluorooctane sulfonamido) acetic acid (MeFOSAA), perfluorodecanoic acid (PFDA), perfluoroheptanoic acid (PFHpA), perfluorononanoic acid (PFNA), perfluoroundecanoic acid (PFUnA), and perfluorododecanoic acid (PFDoA). Concentrations below the LOD were imputed using the standard method of LOD divided by the square root of two (LOD/√2) (Cui et al. 2025).

### 2.4 Diagnosis of Stroke

The primary outcome for this analysis was prevalent stroke. Stroke ascertainment was based on self‐report during the NHANES data collection period. This was determined by an affirmative response to the Medical Condition Questionnaire (MCQ) item: “Has a doctor or other health professional ever told you that you had a stroke?” Participants providing an affirmative response were classified into the “stroke” group, while all others were categorized into the “non‐stroke” group.

### 2.5 Covariates

A range of potential confounders was considered in our analyses to isolate the effects of PFAS on stroke. These included race (five categories: Mexican American, Non‐Hispanic White, Non‐Hispanic Black, Other Hispanic, and Other Race), sex, body mass index (BMI) (three categories: < 25 kg/m^2^, 25–30 kg/m^2^, and ≥ 30.0 kg/m^2^), age, education (three categories: high school or less, some college or AA degree, and college graduate or above), poverty income ratio (PIR) (three categories: ≤ 1 for low, 1–3 for moderate, and ≥ 3 for high), physical activity (PA) (three categories: never, moderate, and vigorous), smoking, alcohol consumption, and history of hypertension, diabetes, and coronary heart disease (CHD) (yes or no). Data on covariates were collected through home interviews, laboratory measurements, and questionnaires to ensure complete adjustment for factors that might influence the study outcomes.

### 2.6 Statistical Analysis

All statistical analyses were performed in accordance with CDC guidelines, accounting for the complex, multistage survey design by incorporating the appropriate NHANES sample weights. Baseline characteristics of the study population were compared by stroke status using the chi‐square test for categorical variables and the *t*‐test for continuous variables. Categorical variables are presented as weighted counts (percentages), and continuous variables as means ± standard deviations (SD). Due to the right‐skewed distribution of serum PFAS concentrations, they were natural log‐transformed (ln‐transformed) to approximate a normal distribution and were analyzed as continuous variables in all regression models (Cui et al. 2025). PFAS concentrations were also categorized into quartiles (Q1–Q4), with the lowest quartile (Q1) serving as the reference category (Wang et al. 2021). Multivariable logistic regression models were used to estimate the association between PFAS exposure (both as ln‐transformed continuous variables and as quartiles) and the odds of stroke. Results are presented as odds ratios (ORs) with 95% confidence intervals (CIs) (Lin et al. 2021). A hierarchical modeling strategy with three levels of covariate adjustment was implemented: Model 1: The crude, unadjusted model; Model 2: Adjusted for key clinical comorbidities (coronary heart disease, hypertension, and diabetes); Model 3: The fully adjusted model, which further adjusted for sociodemographic and lifestyle factors, including age, gender, race/ethnicity, education level, PIR, smoking status, alcohol consumption, physical activity, and BMI.

In addition, this study employed a joint modeling strategy to analyze the mixed exposure effects of multiple PFAS using the WQS model: each PFAS concentration was discretized into quartiles, and the weights were optimized through an adaptive weighting algorithm to construct a combined exposure index, assess the nonlinear dose‐response relationship with stroke risk, and calculate the weighted contribution of each subtype (Weng et al. 2022). Specifically, the concentrations of each PFAS were discretized into equal‐width intervals based on quantile cuts, and the weights of each component were optimized through an adaptive weighting algorithm, ultimately generating a biologically interpretable combined exposure index. Detailed model parameters can be found in the Supplementary Materials (**eMethods: WQS Model**). Subsequently, the dimensionality of the high‐dimensional covariate PFAS data was reduced using PLS‐DA, and the latent variables were extracted and screened for key exposure factors based on variable importance projection (VIP > 1.0) to further validate the discriminative ability of highly weighted factors in the WQS model (Olsson et al. 2021). Additionally, nonlinear associations between key PFAS and stroke risk were assessed using restricted cubic splines (RCS) and tested through subgroup logistic regression and interaction analysis (Wei et al. 2024). All analyses were performed using R 4.3.3, and the significance threshold was set at *p* < 0.05.

### 2.7 Toxicity Analysis and Prediction

To profile the toxicological and pharmacokinetic properties of the key PFAS congeners identified in our epidemiological analysis, we conducted in silico predictions. This was achieved using two publicly available web servers: ProTox 3.0 and admetSAR 3.0. ProTox 3.0 was utilized to predict general toxicological endpoints, while admetSAR 3.0 was employed to specifically evaluate the Absorption, Distribution, Metabolism, Excretion, and Toxicity (ADMET) profiles. The Simplified Molecular Input Line Entry System (SMILES) identifiers for each PFAS congener served as the input for these platforms.

### 2.8 Collection of PFAS Targets

In this study, we employed the following method to construct a target library of PFAS: First, we screened the PFAS most strongly correlated with stroke based on statistical analysis of the NHANES database and then searched for the relevant standardized chemical structures and SMILES identifiers through the PubChem database (Kim et al. 2023). SMILES strings were imported into the ChEMBL and TargetNet databases, while three‐dimensional (3D) structural data were submitted to the PharmMapper database. The screening parameters for each database were set as follows: for ChEMBL, a confidence threshold of ≥ 90% was applied, with the analysis restricted to active compounds; for TargetNet, a probability score of > 0 was required; and for PharmMapper, the search was limited to human proteins with a normalized fit score greater than 0. The targets obtained from the above three databases were standardized using the UniProt database (UniProt Consortium 2023), and the UniProt IDs were converted to standard gene names. Finally, the prediction results from ChEMBL, TargetNet, and PharmMapper were integrated, and after removing the duplicate targets, the combined action target library for the compounds was constructed.

### 2.9 Selection of Stroke—Related Targets Network

In this study, we constructed a set of stroke‐related genes by integrating multiple databases: “Stroke,” “Hemorrhagic stroke,” “Ischemic stroke,” “Apoplexy,” “Hemorrhagic apoplexy,” “Ischemic apoplexy,” “Cerebrovascular accident,” and “CVA” as search terms. The system searched the GeneCards, DisGeNET, and Comparative Toxicogenomics databases (CTD). The screening thresholds were set as follows: GeneCards database entries with a “Relevance Score” exceeding the median, DisGeNET entries with a “GDA score” ≥ 0.1, and CTD entries with an “Inference Score” ≥ 50, in order to identify high‐confidence disease‐associated genes. This approach aligns with methodologies recently employed in studies of environmental toxins and neurological disorders (Cheng et al. 2025). Following consolidation of search outputs from the three databases and elimination of duplicate genes, a definitive panel of genes associated with stroke was established. The target libraries of related PFAS were further mapped and analyzed with the stroke gene set. The compound‐disease targets were visualized using the R language “Venn” package, and key cross‐targets were screened based on the Venn diagram intersections to create a collection of candidate targets for the subsequent mechanistic study.

### 2.10 Assembly of Protein–Protein Interaction (PPI) Networks

Genes shared by both compound targets and disease‐associated profiles were submitted to the STRING database (Szklarczyk et al. 2019) to generate a PPI map, with species limited to Homo sapiens, isolated nodes suppressed, and interaction confidence fixed at the highest threshold (0.9). The network was then exported and visualized in Cytoscape (version 3.10.3) for topological analysis. Key hub genes were identified using the CytoHubba plugin by ranking nodes with four distinct algorithms: Degree, Edge Percolation Component (EPC), Maximum Cluster Centrality (MCC), and Maximum Neighborhood Component (MNC). The top 10 ranked genes from each algorithm were selected for further analysis. The final set of core hub genes was defined as the intersection of these four candidate lists.

### 2.11 Gene Ontology (GO) and Kyoto Encyclopedia of Genes and Genomes (KEGG) Enrichment Analyses

Common target genes were first mapped to Entrez identifiers using the R package “org.Hs.eg.db,” then subjected to Gene Ontology (GO) enrichment and KEGG pathway analyses via the “clusterProfiler,” “enrichplot,”, and “ggplot2” suites. We retained only those GO terms and pathways with both raw and adjusted *p*‐values below 0.05, extracting the top ten GO processes and top thirty KEGG routes by ascending *p*‐value. This workflow revealed the primary roles of hub genes across GO categories—Biological Process (BP), Cellular Component (CC), and Molecular Function (MF)—and illuminated the key signaling pathways in which they participate.

### 2.12 Molecular Docking

A molecular docking approach was employed to study the binding modes of PFAS to core disease targets, assess interaction affinity by predicting binding free energies, and analyze key intermolecular forces. Core targets were designated as receptors, while PFAS were employed as ligands for docking validation. The six hub genes displaying the greatest degree centrality were subsequently chosen for molecular docking with their corresponding PFAS ligands. The 3D structures of the small‐molecule ligands were obtained from PubChem, and 3D Protein Data Bank (PDB) files of the core target proteins were retrieved from the RCSB database based on their UniProt ID (Bittrich et al. 2024). Following preparation in PyMOL 3.1—where water molecules and initial ligands were deleted—the proteins of interest were imported into AutoDock Tools 1.5.6 for protonation, charge assignment, and addition of nonpolar hydrogens. Once the grid box dimensions and genetic algorithm settings were defined, molecular docking was executed via command‐line instructions using AutoDock Vina 1.5.6. Finally, visualization and analysis were performed in PyMOL 3.1 and Discovery Studio 2019.

### 2.13 MD Simulation

To evaluate the stability of the docked protein‐ligand conformations, MD simulations were performed on complexes of key PFAS compounds with their core disease targets using the GROMACS software package (v 2025.1). The stability of these complexes throughout the simulation trajectory was assessed through the analysis of several parameters, including the root mean square deviation (RMSD), radius of gyration (Rg), solvent‐accessible surface area (SASA), and the number of intermolecular hydrogen bonds (HBs). Furthermore, free energy landscape (FEL) plots were generated to visualize the conformational sampling and energetic stability of the protein‐ligand complexes. The script for FEL generation was adapted from an open‐source project by Charles Hahn (https://github.com/CharlesHahn/DuIvy/blob/master/sources/other/PCA_FEL/xpm2png.py). Finally, to quantify the binding affinity, the binding free energy for each complex was calculated over the final 30 ns of the simulation trajectory using the gmx_Molecular Mechanics/Generalized Born Surface Area (MM/GBSA) tool (Dash et al. 2021).

## 3 Results

### 3.1 Baseline Characteristics

Using NHANES data, we evaluated 8,081 participants (Supplementary **eTable** **2** **, Supplementary** **eFigure** **1**), of whom 3,942 were men and 4,139 were women. Individuals with a history of stroke were, on average, older (67.8 ± 13.2 years) and had higher body‐mass indices (29.5 ± 6.5 kg/m^2^) than their non‐stroke counterparts. The male‐to‐female ratio did not differ significantly between groups, yet numerous baseline characteristics—including ethnicity, BMI category, hypertension, diabetes, coronary heart disease, alcohol intake, educational level, poverty‐income ratio, and physical activity—showed significant disparities between the stroke and non‐stroke cohorts.

### 3.2 Association Between PFAS and Stroke Risk

To further investigate the association between PFAS monomers and stroke risk, we conducted PFAS‐stratified logistic regression analysis. The results indicated that, after adjusting for all covariates, increased concentrations of six PFAS were associated with an elevated risk of stroke onset. Specifically, the following PFAS monomers showed significant associations with stroke risk: PFOS [Odds Ratio (OR) = 1.59, 95% CI: 1.09–2.31], N‐ETFOSAA (OR = 1.53, 95% CI: 1.18–1.97), N‐MEFOSAA (OR = 2.00, 95% CI: 1.30–3.08), PFHPA (OR = 1.55, 95% CI: 1.17–2.05), PFUA (OR = 1.57, 95% CI: 1.04–2.38), and PFDO (OR = 1.57, 95% CI: 1.22–2.03) (**eTable** **3**).

**FIGURE 1:**
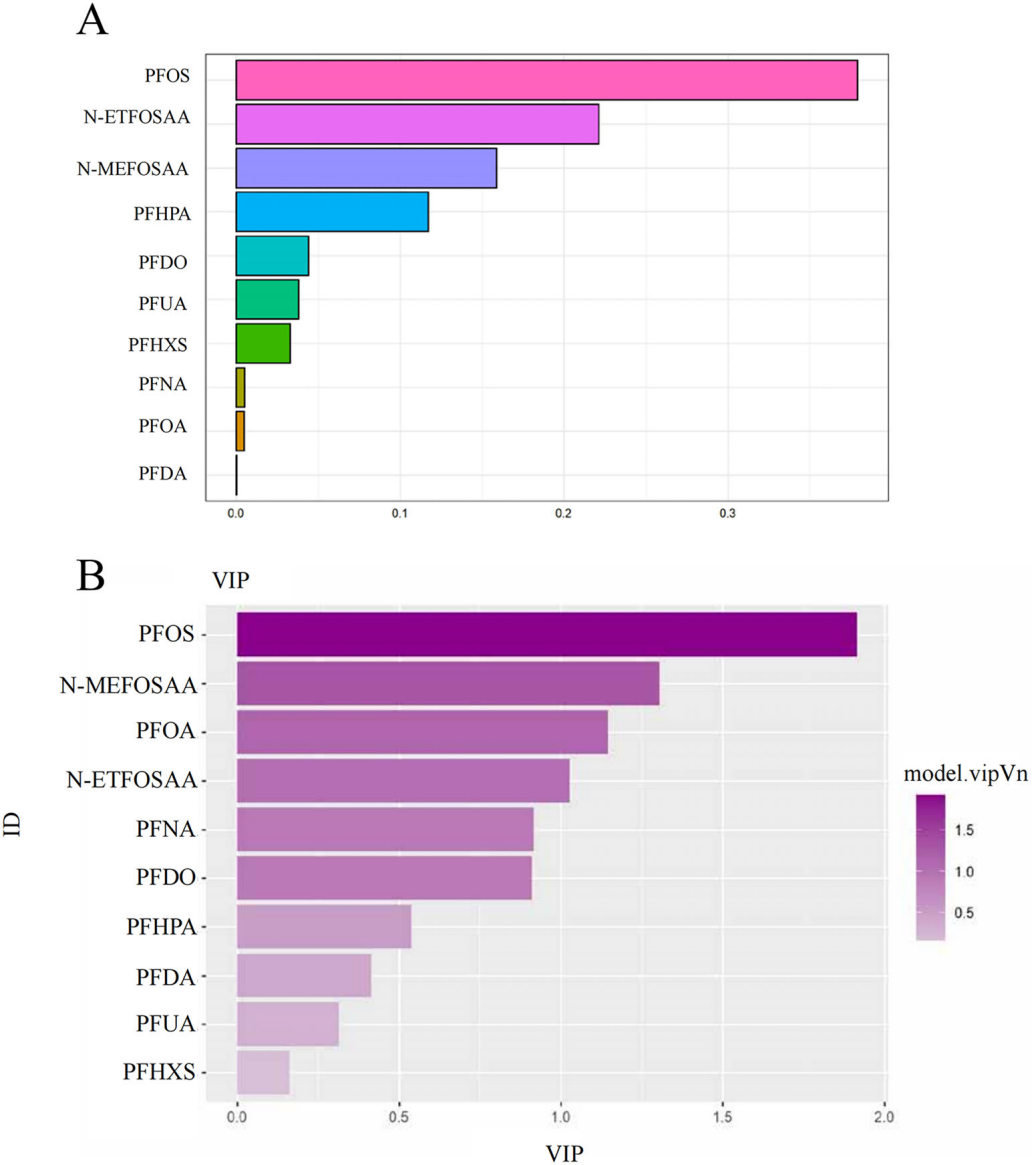
A combined approach using WQS and PLS‐DA to evaluate the relationship between PFAS exposure and the risk of stroke. **(A)** Screening of key exposed monomers in PFAS using PLS‐DA modeling. **(B)** The WQS model assigns weights to serum PFAS levels, indicating a positive association with the risk of stroke. This model was adjusted for factors including age, BMI, education, ethnicity, gender, alcohol consumption, smoking, CHD, hypertension, diabetes, PA, and PIR.

Given the potential for co‐exposure patterns and multicollinearity among PFAS monomers, these issues cannot be fully addressed by conventional regression models. Therefore, the present study employed both WQS regression and PLS‐DA models to analyze the mixture effects. WQS results demonstrated a significant positive association between cumulative PFAS exposure and stroke risk (OR = 1.027, 95% CI: 1.017–1.036; p < 0.01), with PFOS contributing the highest weight at 0.379, indicating its dominant role (Figure 1A). To further validate the key factors, we applied the PLS‐DA model to identify the PFAS monomers that significantly contributed to the classification of stroke patients versus non‐stroke patients using Variable Importance in Projection (VIP). The results showed that PFOS had the highest discriminatory power, with a VIP score greater than 1.5 (Figure 1B). Consistency analysis between the two models demonstrated that PFOS's high weight in mixed exposures (WQS) and strong discriminatory power (PLS‐DA) collectively supported its central association with stroke risk.

### 3.3 Dose‐response Relationship and Subgroup Analysis Between PFOS and Stroke Risk

The dose‐response relationship between circulating PFOS concentration and the odds of stroke was modeled using a fully adjusted restricted cubic spline (RCS) (Supplementary eFigure 2A). While the overall test for a non‐linear association did not reach statistical significance (*P* for non‐linearity = 0.068), the spline curve visually suggested a potential threshold effect. The odds of stroke remained relatively stable at lower PFOS levels, followed by a monotonic increase at higher concentrations. This inflection occurred at an ln‐transformed PFOS concentration of approximately 1.4.

To formally test the threshold effect suggested by the RCS model, a segmented logistic regression was performed (Supplementary eFigure 2B). This analysis identified a significant breakpoint at a log_10_‐transformed PFOS concentration of 1.1. Concentrations exceeding this threshold were significantly associated with higher odds of stroke (OR = 1.22, 95% CI: 1.04–1.44, *p* = 0.017). Consistent with these non‐linear and threshold analyses, treating ln‐transformed PFOS as a continuous variable in a standard logistic regression model also revealed a significant positive association with stroke (OR = 1.19, 95% CI: 1.03–1.38, *p* = 0.015).

In addition, to test the robustness of the PFOS–stroke relationship across diverse demographic and clinical strata, we performed subgroup analyses stratified by age, BMI, sex, ethnicity, hypertension, diabetes, CHD, smoking, alcohol, educational level, PIR, and PA. Elevated PFOS concentrations remained significantly linked to stroke risk in every subgroup examined (*p* < 0.05), with the strongest association observed among adults aged 60 years or older (Supplementary **eFigure** **3**). These results underscore the consistency of the PFOS–stroke connection across varied population segments.

### 3.4 PROTox and admetSAR Prediction of Toxicity of Chemicals

Predictions of PFOS toxicity, as indicated by the PROTox and admetSAR databases, suggest its potential to cause organ toxicity (e.g., respiratory toxicity) and disrupt the blood‐brain barrier (BBB) (Supplementary **eTables** **4, 5**). However, predictions regarding neurotoxicity vary across platforms (e.g., admetSAR indicates activity, whereas PROTox does not). Given the potential limitations of computational prediction models stemming from variations in algorithms and training sets, we examined the extensive experimental literature. Numerous in vivo and in vitro studies have demonstrated that PFOS exposure impairs learning and memory functions in experimental animals, induces neuronal apoptosis, and compromises blood‐brain barrier integrity (Zhang et al. 2023, Miralles‐Marco and Harrad 2015). This robust body of experimental evidence collectively supports the hypothesis that PFOS induces neurotoxicity. Therefore, we performed a network toxicology analysis to further investigate whether PFOS elevates the risk of stroke.

### 3.5 Target Retrieval

**FIGURE 2:**
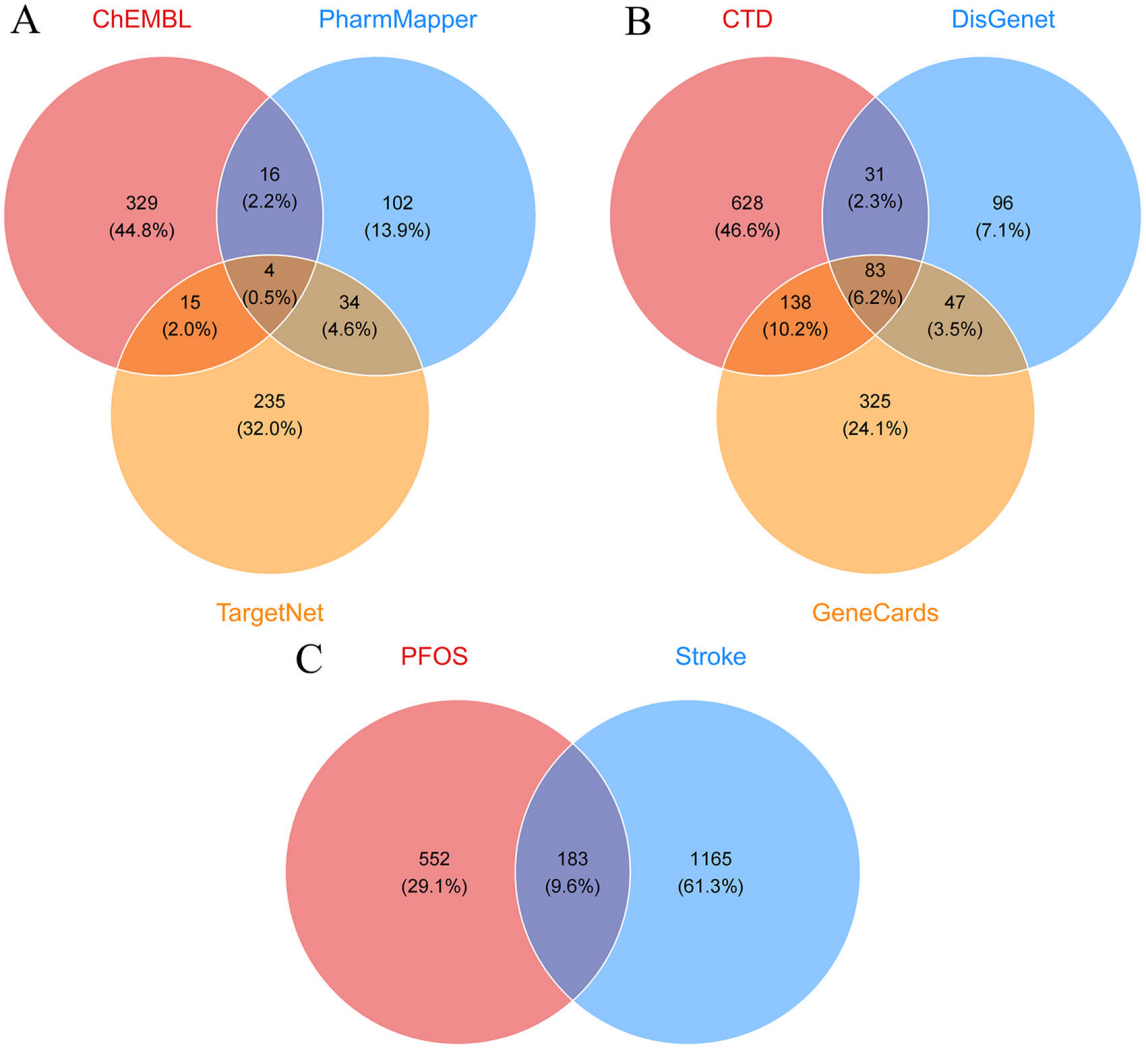
Venn diagrams of intersecting genes. **(A)** Target collection for PFOS, **(B)** Target collection for stroke, and **(C)** A Venn diagram illustrating the overlapping targets between PFOS and stroke.

To delineate PFOS's molecular targets, we pooled prediction results from ChEMBL, TargetNet, and PharmMapper, uncovering 735 candidate genes (Figure 2A). Concurrently, we assembled stroke‐associated genes from GeneCards (593 entries), DisGeNET (371 entries), and the Comparative Toxicogenomics Database (881 entries). After eliminating redundancies, this yielded 1348 distinct stroke‐related genes (Figure 2B). By intersecting this set with the PFOS target list, we identified 183 common genes (Figure 2C, Supplementary **eTable** **6**), highlighting their potential involvement in PFOS‐induced stroke.

### 3.6 PPI Network of Shared Genes and Discovery of Core Targets

**FIGURE 3:**
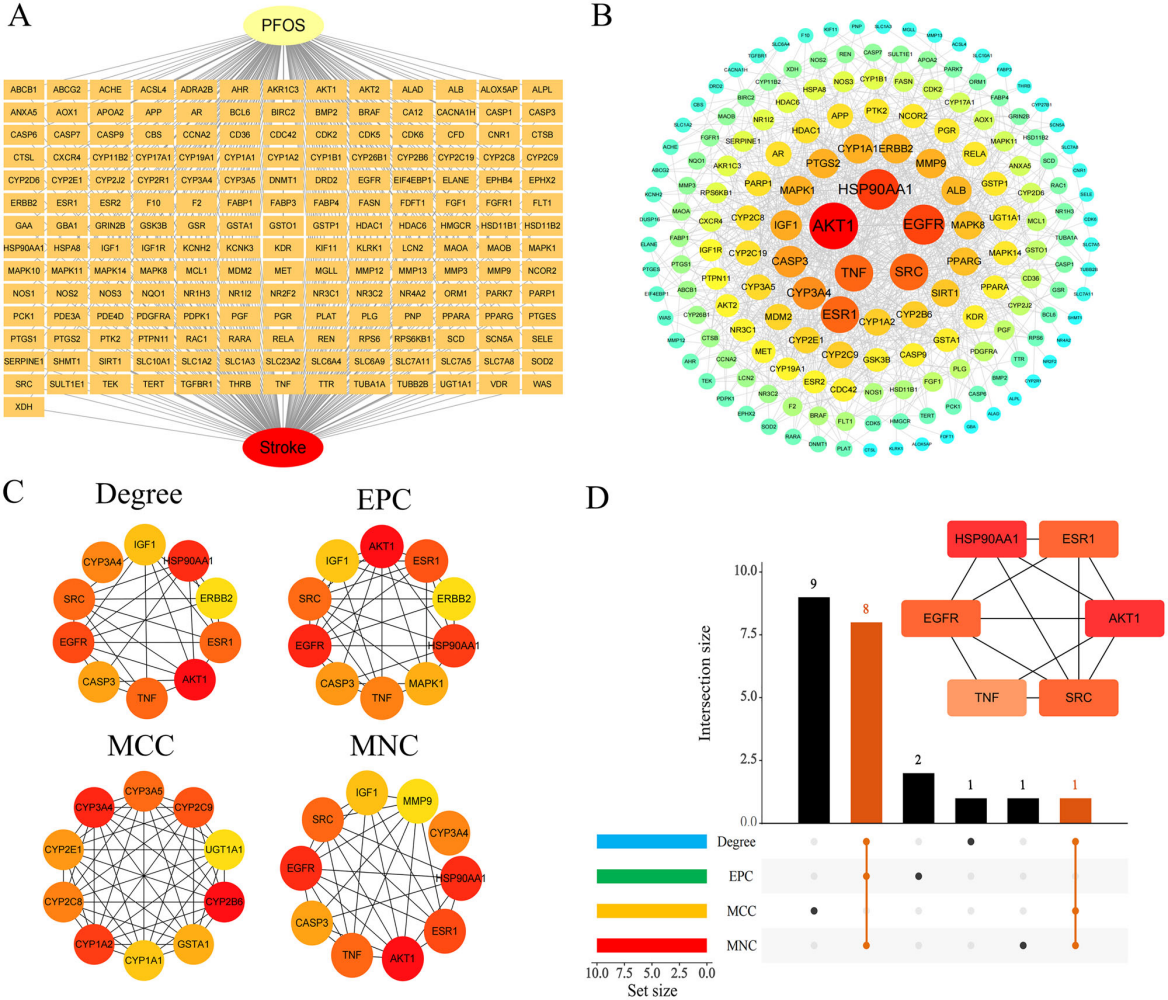
Identification of key and potential targets through the use of Cytoscape and the STRING database. **(A)** PFOS‐target‐stroke network diagram, **(B)** The PPI network is visualized and analyzed in detail using Cytoscape 3.10.3. Nodes are represented with varying colors and sizes based on their degree values, where darker hues and larger circles signify stronger interactions; **(C)** The top 10 genes with the highest connectivity in the PPI network, as identified by Cytoscape; and **(D)** An UpSet plot displaying the overlap of six hub genes obtained from the Degree, EPC, MCC, and MNC algorithms within the CytoHubba plugin.

To examine how the overlapping PFOS‐stroke targets interrelate, we used the STRING database to generate a protein–protein interaction network, setting a confidence score threshold of ≥ 0.9. This approach resulted in 183 nodes, 907 edges, and an average node degree of 9.91 (Supplementary **eFigure** **4**). Subsequently, we exported TSV files from STRING and imported them into Cytoscape 3.10.3 to visualize the protein interaction network, creating a compound‐target‐stroke network graph (Figure 3A) and an optimized, intuitive PPI network graph (Figure 3B). Nodes with the highest degree values are marked in dark red, with the color progressively fading as the degree value diminishes. The size of each node corresponds to its degree, while the links between the nodes indicate protein interactions. Additionally, we used the CytoHubba plug‐in in Cytoscape and analyzed the network with four algorithms: Degree, EPC, MCC, and MNC. This analysis identified six core gene targets: AKT1, HSP90AA1, EGFR, SRC, ESR1, and TNF (Figure 3C**, D**). These genes are well‐established for their critical roles in various cellular functions. This visualization method provides a comprehensive overview of the interactions among key targets, offering important insights that can guide further investigation into the molecular mechanisms connecting PFOS to stroke.

### 3.7 GO and KEGG Enrichment Analysis

**FIGURE 4:**
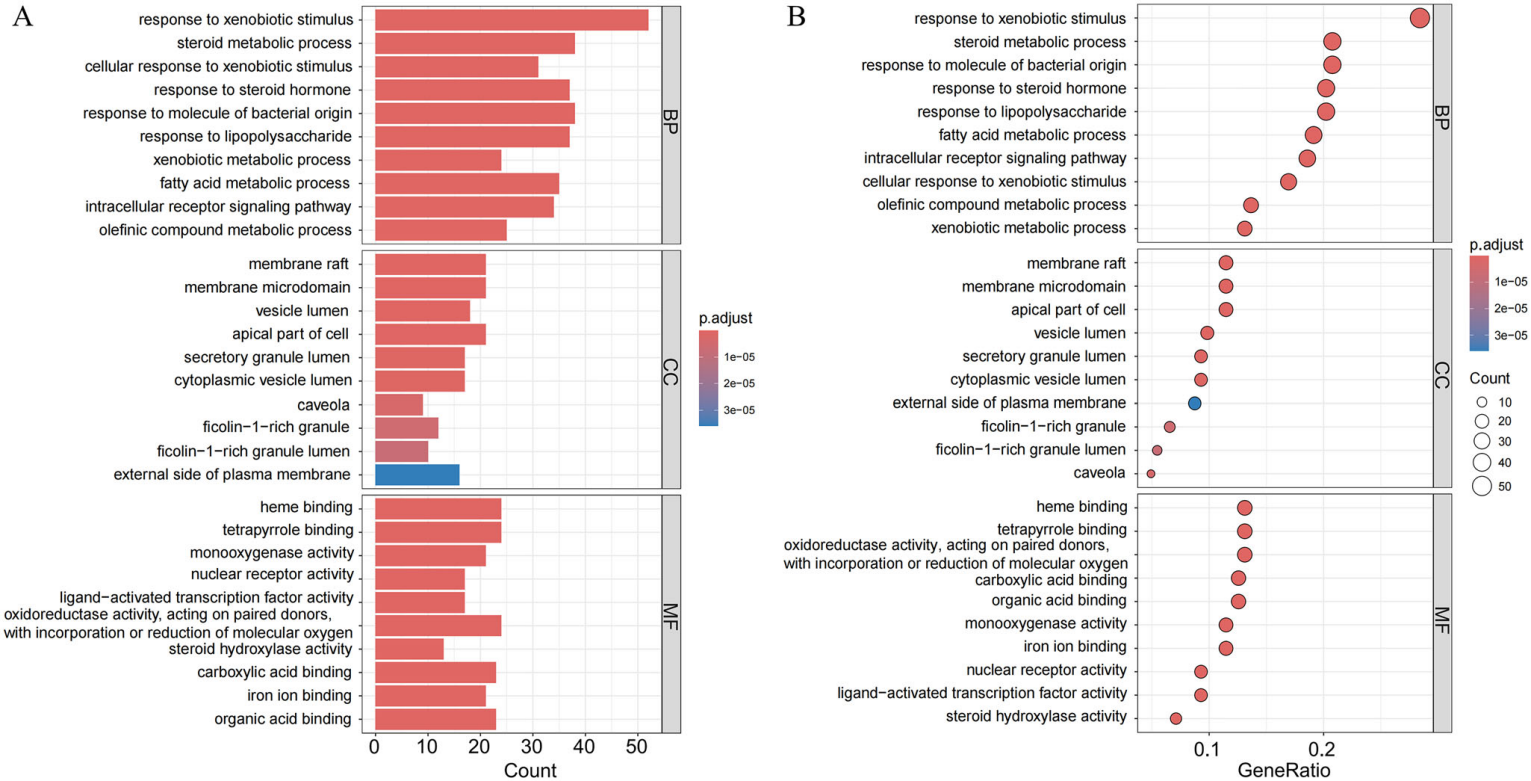
GO enrichment analysis of potential targets (top 10). **(A)** This histogram displays the top 10 terms with the highest enrichment levels among 183 potential targets across Gene Ontology categories—BP, CC, and MF—sorted by FDR value. Lower FDR values indicate greater statistical significance for enrichment. The height of each bar represents the number of genes associated with that term, reflecting the degree of enrichment within that category. These highlighted terms emphasize key biological processes, cellular components, and molecular functions potentially affected by PFOS exposure, and **(B)** A bubble chart presents the count and −log10 (*p*‐value) for the top 10 enriched entries in each GO category (BP, CC, and MF) associated with hub genes. The bubble size reflects gene expression levels, while the color saturation indicates the significance of enrichment.

**FIGURE 5:**
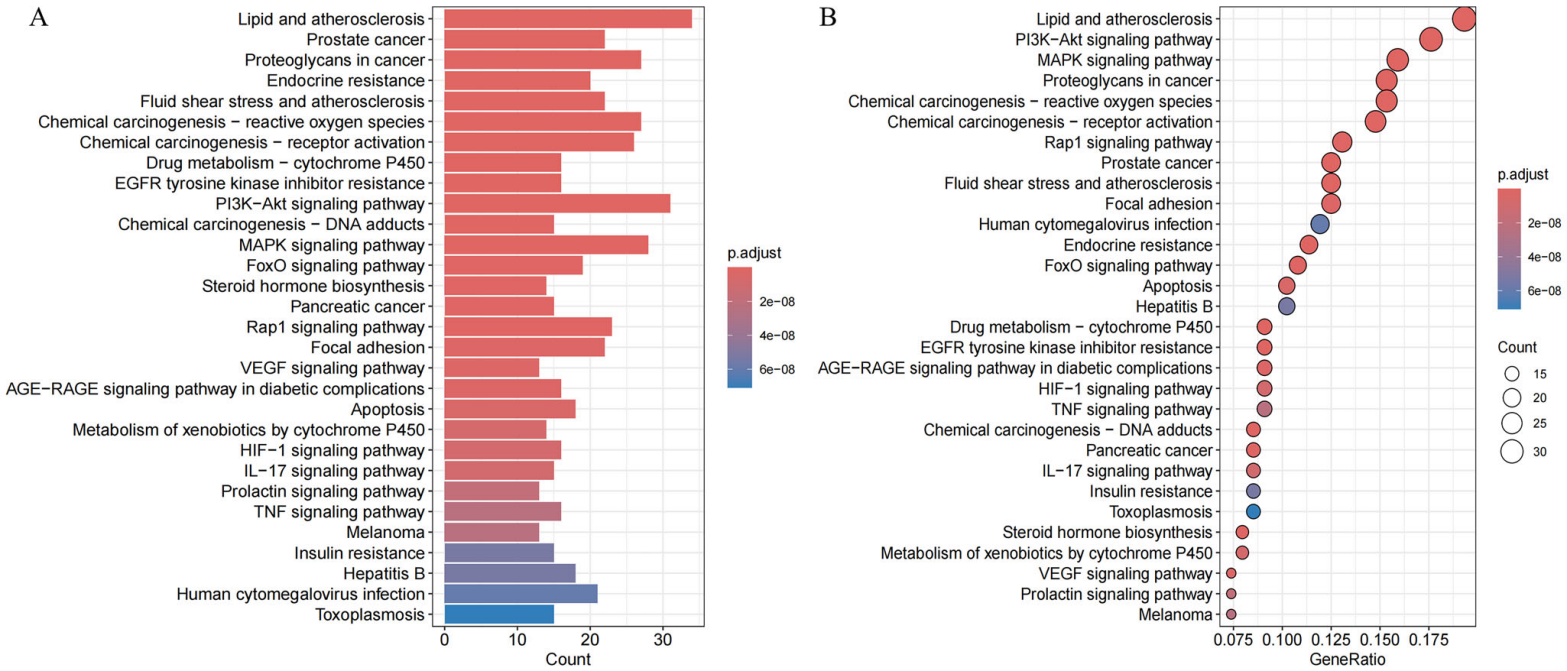
KEGG enrichment analysis of potential targets (top 30). **(A)** The histogram depicts the frequency and significance of enrichment across different pathways. The bar length represents the number of genes involved, reflecting both the enrichment score and significance level. Taller bars correspond to a higher gene count and stronger enrichment and **(B)** The bubble chart visualizes the top 30 enriched KEGG signaling pathways, ordered by decreasing FDR values. Each bubble corresponds to a specific pathway, with its size indicating the number of enriched genes. The color intensity signifies the enrichment significance, where deeper red shades reflect greater statistical relevance.

To systematically elucidate the biological mechanism of PFOS‐induced stroke, we performed GO functional annotation and KEGG pathway enrichment analysis of the cross‐target genes using the “clusterProfiler” software package in R. Applying a stringent False Discovery Rate cutoff of 0.05, we then plotted the ten GO terms in each category exhibiting the smallest False Discovery Rate (FDR) values (Figure 4A**, B**). The results of the multidimensional analysis of GO revealed that genes were significantly enriched in response to xenobiotic stimulus at the BP level and significantly enriched in membrane raft, membrane microdomain, and apical part of the cell at the CC level. At the MF level, genes were significantly enriched in heme binding and tetrapyrrole binding. Additionally, KEGG pathway analysis was conducted using hierarchical clustering, and the 30 pathways with the lowest FDR values were selected to construct the categorized bar graph and bubble diagram (Figure 5A**, B**). The KEGG pathway analysis revealed that these genes were mainly associated with Lipid and Atherosclerosis, the PI3K‐Akt signaling pathway, the MAPK signaling pathway, Proteoglycans in cancer, and Chemical carcinogenesis through reactive oxygen species, with lipid and atherosclerosis being the most significant. According to the KEGG database's classification framework, the key pathways were systematically organized into five functional groups: Organismal Systems, Metabolism, Human Diseases, Environmental Information Processing, and Cellular Processes (Supplementary **eFigure** **5**). Within the Organismal Systems category, genes were predominantly enriched in the relaxin signaling pathway and the IL‐17 signaling pathway. In the Metabolism category, the primary enrichment was observed in drug metabolism via cytochrome P450. For the Human Diseases category, genes were notably enriched in lipid and atherosclerosis, chemical carcinogenesis through reactive oxygen species, and proteoglycans in cancer. In the Environmental Information Processing category, the genes were chiefly enriched in the PI3K‐Akt and MAPK signaling pathways. Finally, in the Cellular Processes category, genes showed significant enrichment in focal adhesion and apoptosis. Therefore, the pathological process of PFOS‐induced stroke is driven by the interaction of multiple biological mechanisms, including oxidative stress, inflammatory dysregulation, metabolic imbalance, and apoptosis.

### 3.8 Molecular Docking for PFOS and Core Target Proteins of Stroke

**FIGURE 6:**
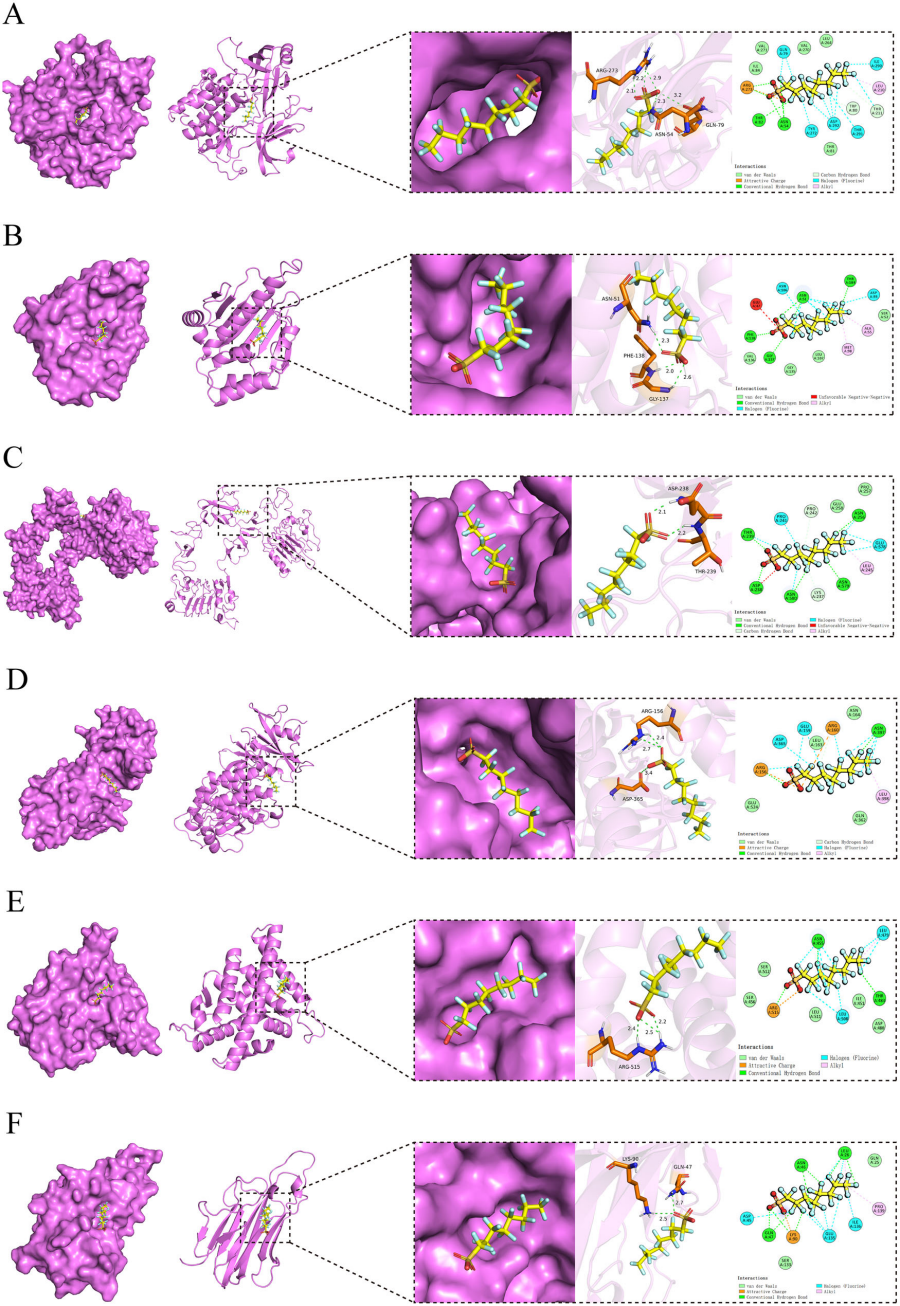
Molecular docking of PFOS with six core targets in stroke. **(A)** PFOS and AKT1, binding energy −9.8 kcal/mol; **(B)** PFOS and HSP90AA1, binding energy −7.8 kcal/mol; **(C)** PFOS and EGFR, binding energy −6.7 kcal/mol; **(D)** PFOS and SRC, binding energy −8.6 kcal/mol; **(E)** PFOS and ESR1, binding energy −7.0 kcal/mol; and **(F)** PFOS and TNF, binding energy −5.7 kcal/mol.

Molecular docking revealed the interaction of PFOS with six hub genes. PFOS exhibits strong binding to all six genes, with binding energies varying between −5.7 and −9.8 kcal/mol. According to the criteria, negative binding energies indicate binding activity, with values below −5.0 kcal/mol suggesting strong binding, and lower values indicating stronger interactions. Notably, AKT1 exhibited the highest binding affinity of −9.8 kcal/mol, due to the formation of a salt bridge between the ligand and ARG273 on the protein, as well as six hydrogen bonds involving THR82, ASN54, and ARG273, and the ligand. Additionally, van der Waals forces between amino acids further stabilized the binding. SRC exhibits the second strongest binding affinity, at −8.6 kcal/mol. A more detailed examination of intermolecular interactions revealed that PFOS primarily associates with target proteins via non‐covalent interactions. These include typical hydrogen bonds, carbon‐hydrogen bonds, and alkyl interactions (**Figure** **6**). The docking results were analyzed using PyMOL 3.1 for 3D conformational simulations and Discovery Studio 2019 for 2D interaction visualization.

### 3.9 MD Simulation

**FIGURE 7:**
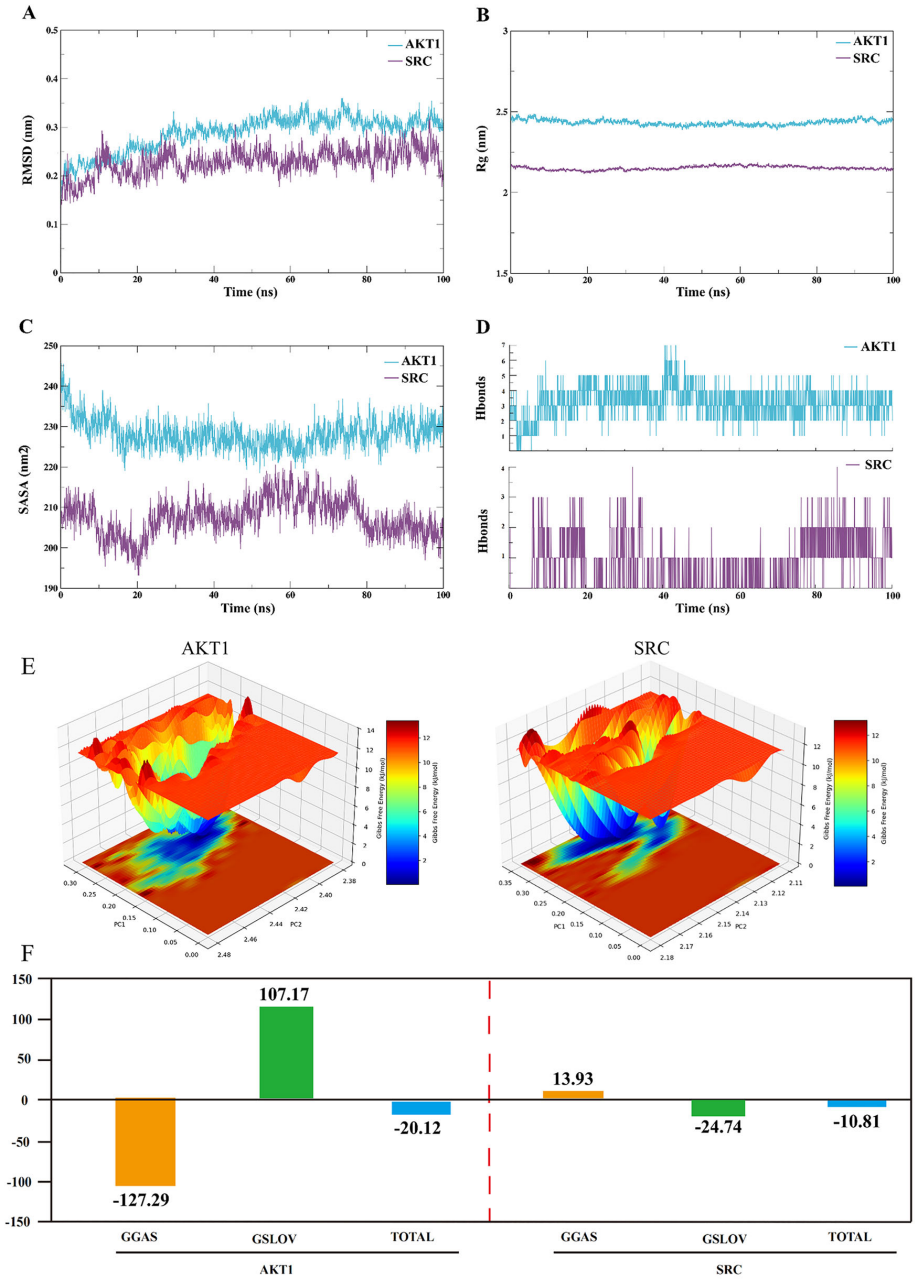
Results of molecular dynamics simulation. **(A)** RMSD values of the two complexes, **(B)** Rg values of the two complexes, **(C)** SASA values of the two complexes, **(D)** Number of hydrogen bonds in the two complexes, **(E)** Free energy landscapes of two complexes, and **(F)** Binding free energies of the two complexes. **Abbreviations**: GGAS, gas‐phase interaction energy; GSOLV, solvation free energy; Total, sum of GGAS and GSOLV.

To assess the binding stability of the ligand‐protein complexes, the two PFOS‐target complexes with the highest predicted binding affinities, namely PFOS‐AKT1 and PFOS‐SRC, were selected for MD simulations. The RMSD values for both systems stabilized at 0.3111 ± 0.0142 Å and 0.2447 ± 0.0221 Å, respectively, indicating that conformational stability was achieved (Figure 7A). The Rg values remained consistent at 1.8537 ± 0.1977 nm and 2.0953 ± 0.2632 nm, confirming the structural compactness of the complexes (Figure 7B). Analysis of the SASA yielded average values of 206.3925 ± 3.6756 nm^2^ and 228.9897 ± 2.7075 nm^2^, respectively, reflecting stable yet distinct solvent exposure profiles (Figure 7C). Furthermore, hydrogen bond analysis revealed that the PFOS‐AKT1 and PFOS‐SRC complexes formed a maximum of 7 and 4 hydrogen bonds, respectively, underscoring the significant contribution of these interactions to complex stability (Figure 7D). The FEL over the final 30 ns of the simulation revealed the presence of multiple low‐energy states, which further corroborates the stability of the complexes (Figure 7E). Finally, binding free energies (∆Gbind) were calculated using the MM/GBSA method. The calculated ∆Gbind values for PFOS‐AKT1 and PFOS‐SRC were −20.12 and −10.81 kcal/mol, respectively (Figure 7F).

## 4 Discussion

Widespread human exposure to PFAS is nearly ubiquitous due to their environmental persistence and extensive use in consumer and industrial products (Panieri et al. 2022). While research has linked PFAS mixtures to CVD and implicated specific inflammatory pathways (Mao et al. 2024), the association between individual PFAS congeners and the risk of stroke has remained inconsistent and poorly understood. Addressing this knowledge gap, our study employed a multi‐pronged approach combining epidemiological analysis of a nationally representative US population with in silico toxicological and network pharmacology methods. Our primary epidemiological finding is that among 12 PFAS congeners, exposure to PFOS was most robustly and significantly associated with prevalent stroke, an association that persisted after comprehensive covariate adjustment. Based on databases such as ChEMBL, TargetNet, PharmMapper, GeneCards, DisGeNET, and CTD, as well as network assessment tools, 183 potential targets associated with PFOS‐induced stroke were identified in this study. The interaction network of these targets was constructed using the STRING platform and Cytoscape software, and the six core hub genes of PFOS‐induced stroke, including AKT1, HSP90AA1, EGFR, SRC, ESR1, and TNF, were further screened and validated by four algorithms of Degree, EPC, MCC, and MNC, as well as molecular docking. Molecular docking analysis showed that the core target proteins formed stable binding with PFOS with binding energies ranging from −5.7 to −9.8 kcal/mol, suggesting a potent affinity. These hub genes provide a key theoretical basis for elucidating the molecular mechanism of PFOS‐induced stroke.

While the structure‐activity relationships among PFAS congeners remain incompletely elucidated, these relationships offer a critical perspective for interpreting our epidemiological findings (Liu et al. 2025). The differential effects observed among congeners are primarily attributed to their unique physicochemical properties, which govern their bioavailability, pharmacokinetics, and toxicokinetics (Amstutz et al. 2022). Specifically, PFOS—an eight‐carbon perfluoroalkyl sulfonate with a sulfonic acid head group—exhibits greater biological activity and a longer half‐life compared to carboxylic acid analogues such as PFOA or shorter‐chain substitutes, resulting in prolonged systemic exposure (Wen et al. 2024). The length of the perfluorinated carbon chain (C ≥ 7) is a critical determinant of both bioaccumulation potential and toxicity. The octa‐carbon chain of PFOS enables it to more effectively disrupt the lipid bilayer of neurovascular units, compromising blood‐brain barrier integrity; this finding is consistent with our toxicity prediction results (Leuthner et al. 2025, Feng et al. 2023, Hamid et al. 2023). Critically, our molecular docking and kinetic simulations elucidate the structural basis of this structure‐activity relationship. The unique molecular architecture of PFOS, characterized by its fluorinated tail and the size and electronegativity of its polar head group, facilitates high‐affinity binding to core targets such as AKT1 (binding energy: −9.8 kcal/mol). This binding is stabilized within the target pocket through optimal hydrogen bonding (e.g., with ARG273 and THR82 of AKT1) and hydrophobic interactions. In contrast, shorter‐chain or differently functionalized PFAS congeners exhibit markedly lower binding affinities. This differential binding affinity provides a mechanistic explanation for why PFOS carries the highest weighting in the Weighted Quantile Sum (WQS) mixed exposure model. Consequently, the dominant role of PFOS in the observed stroke risk association is fundamentally rooted in its unique chemical structure, which simultaneously confers exceptional environmental persistence and specific molecular disruption capabilities (Amstutz et al. 2022).

AKT1, a pivotal kinase downstream of the PI3K/AKT signaling pathway, is a central regulator of cell survival through the inhibition of apoptosis (Manning and Toker 2017). Specifically, phosphorylated AKT1 significantly promotes neuronal survival and induces cerebral ischemic tolerance post‐stroke by enhancing its downstream effectors, PRAS40 and mTOR; this mechanism has been validated in two independent studies using distinct models (Li et al. 2017, Xie et al. 2013). However, PFOS exposure reportedly induces damage in supporting cells via AKT1/2‐mediated disruption of F‐actin and microtubule organization, indicating potential pathway‐specific vulnerabilities (Gao et al. 2024). Additionally, HSP90AA1, a member of the HSP90 family, is a critical heat shock protein responsible for maintaining cellular homeostasis. In the context of stroke, genetic variants in the HSP90AA1 gene are associated with stroke risk, recurrence risk, and modulation of the inflammatory response (Kobzeva et al. 2024). Although a direct link between PFOS exposure and HSP90AA1 regulation has not been established, PFOS is known to activate PPAR‐α pathways implicated in oxidative stress and neurotoxicity—processes where HSP90AA1 has established regulatory roles (Gou et al. 2024). Furthermore, the transmembrane receptor EGFR is abundantly expressed in the developing brain and neurogenic regions, with lower expression levels observed in the adult brain. Animal studies have revealed that pharmacological blockade of EGFR markedly reduces the incidence of intracranial aneurysm rupture and downregulates the mRNA expression of endoplasmic reticulum stress markers and inflammatory cytokines in cerebral arteries (Ishiguro et al. 2024). PFOS exposure has been observed to downregulate microRNAs that target EGFR, which may lead to its upregulation, potentially altering vascular responses and impairing post‐stroke tissue repair (Xu et al. 2020). SRC, a non‐receptor tyrosine kinase, initiates multiple signaling pathways in various types of vascular smooth muscle cells. SRC kinase functions as a critical intermediary linking the PI3K/AKT and MAPK pathways, both of which regulate inflammation and apoptosis in the context of stroke (Li et al. 2023). Intriguingly, PFOS‐induced miRNA suppression (e.g., miR‐101‐3p) may simultaneously enhance both EGFR expression and the activation of the downstream SRC/PI3K/MAPK pathway (Xu et al. 2020). Beyond these signaling cascades, polymorphisms in ESR1 are associated with stroke risk through the modulation of estrogen receptors. ESR1, which encodes estrogen receptor α, is widely expressed in the central and cardiovascular systems. Genetic polymorphisms in the ESR1 gene have been shown to influence the biological effects of estrogen by modulating ESR1 expression levels and are linked to stroke risk (Clegg et al. 2017); concurrently, the endocrine‐disrupting properties of PFOS may exacerbate cerebrovascular risks by interfering with the estrogen pathway (Kobzeva et al. 2024). TNF, a pleiotropic cytokine, is well known for its pivotal role in inflammation. TNF‐α is a key driver of chronic post‐stroke neuroinflammation, exacerbating neuronal damage by mediating signaling pathways via its receptors, TNFR1 and TNFR2, which are implicated in apoptosis, inflammatory cell infiltration, and BBB disruption (Alsbrook et al. 2023). One study reported that PFOS exposure was associated with a significant decrease in TNF‐α and IL‐10 levels, suggesting that PFOS may modulate immune function by suppressing inflammatory responses (Siwakoti et al. 2024). Thus, PFOS exposure significantly influences TNF‐α levels, potentially affecting stroke‐related inflammatory mechanisms. Collectively, these interconnected pathways highlight the multifaceted interplay between PFOS exposure and stroke pathophysiology, encompassing kinase regulation, stress response modulation, receptor signaling, and inflammatory mediation.

Furthermore, GO and KEGG pathway analyses were conducted on the genes common to both PFOS and stroke. These analyses revealed that PFOS may contribute to stroke pathogenesis primarily via a synergistic network of pathways, including lipid metabolism, atherosclerosis, and the PI3K/AKT and MAPK signaling pathways. The lipid metabolism and atherosclerosis pathways represent a core pathological foundation for stroke and are closely linked to PFOS‐induced dysregulation of lipid metabolism, oxidative stress, inflammation, and endocrine function (Osorio‐Yáñez et al. 2021, Liu et al. 2007, Salihovic et al. 2020, Kahn et al. 2020). Collectively, these molecular mechanisms synergistically promote the formation and rupture of atherosclerotic plaques, ultimately elevating stroke risk. PFOS is hypothesized to induce lipid dysmetabolism by disrupting the PPAR‐α and cholesterol efflux transporter pathways (Bijland et al. 2011, Lind and Lind 2020, Fletcher et al. 2013). Moreover, PFOS can disrupt human lipid homeostasis through PPARα activation, HNF4α pathway inhibition, and the dysregulation of bile acid synthesis and transport; consequently, prolonged exposure may increase the risk of atherosclerosis and stroke (Fragki et al. 2021). The PI3K/AKT signaling cascade is a central regulator of brain homeostasis and a critical component in the molecular pathophysiology of stroke (Liu et al. 2025, Chen et al. 2020). This pathway is crucial for angiogenesis and oxidative stress regulation during stroke, as it not only governs cell survival, proliferation, and metabolism but also counteracts processes such as endothelial cell apoptosis, impaired vascular repair, and metabolic dysfunction (Gu et al. 2022, Hawkins and Davis 2005). Inhibition of the PI3K/AKT signaling pathway has been shown to increase neuronal susceptibility to excitotoxicity (Delgado‐Esteban et al. 2007). PI3K/AKT is widely distributed throughout the body, and its downstream effectors perform multiple biological functions (Liu et al. 2025). For instance, in astrocytes, PFOS has been demonstrated to mediate the secretion of proinflammatory cytokines, such as IL‐1β, via the PI3K/AKT/NF‐κB pathway, thereby implicating this cascade in PFOS‐induced neuroinflammation (Li et al. 2024). Notably, in addition to the aforementioned neuroinflammatory mechanisms, a direct link between PFAS exposure, platelet hyperactivation, and thrombosis constitutes a critical pathway contributing to an elevated stroke risk (Meneguzzi et al. 2021, Lin et al. 2022). Studies have demonstrated that PFAS can directly bind to the GPIbα receptor on platelet membranes, thereby initiating an intracellular signaling cascade that results in calcium mobilization and subsequent activation of the Akt pathway. This cascade, in turn, induces platelet aggregation, fosters a procoagulant state, and drives the formation of neutrophil extracellular traps (NETs), ultimately accelerating thrombus growth and elevating the risk of ischemic stroke (Liu et al. 2025). Collectively, this mechanism establishes a direct causal pathway between PFAS exposure, platelet hyperactivation, and thrombogenesis, thereby offering a novel mechanistic explanation for PFAS‐associated cardiovascular pathologies. Analogous to the PI3K/AKT pathway, the MAPK signaling pathway plays a critical regulatory role in both ischemic and hemorrhagic cerebrovascular diseases (Sun and Nan 2016, Xie et al. 2019). This pathway regulates fundamental biological processes, including cell proliferation, differentiation, migration, and apoptosis (Imajo et al. 2006). The MAPK family is composed of three primary subfamilies: c‐Jun N‐terminal kinase (JNK), p38 mitogen‐activated protein kinase (p38), and extracellular signal‐regulated kinase (ERK) 1/2. These subfamilies are differentially activated: JNK and p38 are predominantly activated by inflammatory signals and cellular stress, whereas ERK1/2 activation is primarily dependent on growth factors and cytokines (Slattery et al. 2018). A study in zebrafish embryos revealed that PFOS exposure induced an increase in ROS production and an upregulation of MAPK component expression—particularly JNK and p38—resulting in oxidative stress and apoptosis (Shi and Zhou 2010). Furthermore, an animal study demonstrated that perinatal PFOS exposure, when combined with a high‐fat diet, altered calcium signaling and the MAPK pathway in the brain, potentially contributing to neurobehavioral changes and an increased risk of stroke (Hmila et al. 2024).

Notably, subgroup analyses revealed significant heterogeneity in effect sizes across gender and racial/ethnic groups. For instance, the association between PFOS exposure and stroke risk was more pronounced in males, while populations such as Mexican Americans and non‐Hispanic whites exhibited higher odds ratios (ORs) compared to other groups. These disparities may be attributable to several factors. First, genetic variations across populations—particularly polymorphisms related to lipid metabolism, inflammatory responses, and vascular function—may influence PFAS pharmacokinetics (Pomazal et al. 2024). Additionally, variations in dietary habits, occupational exposures, and social behaviors across ethnic groups may lead to variable cumulative exposure levels (Ding et al. 2023, Liddie et al. 2023). Furthermore, gender‐specific effects warrant attention, particularly because PFOS is a well‐established endocrine disruptor that may exert such effects by interfering with estrogen signaling pathways. For example, the absence of estrogen's potential cardioprotective effects in men could contribute to a more pronounced increase in risk (Wu et al. 2024). Moreover, differences in body fat distribution and PFAS metabolism rates between genders may also explain these risk disparities (Haug et al. 2023, Roth et al. 2021). In summary, the observed heterogeneity in the PFOS‐stroke risk association across genders and ethnicities likely results from the complex interplay of multiple factors, including genetic susceptibility, sociodemographic factors, baseline health status, and PFOS's inherent endocrine‐disrupting properties. These findings underscore the critical importance of stratified population analysis and the in‐depth investigation of effect‐modifying factors in future environmental health research.

Although this study fills a mechanistic gap in the association of PFAS with stroke exclusively, several limitations remain. The most significant limitation is the absence of in vivo experiments to confirm the effect of PFOS on stroke, which would support the identification of core targets and effector pathways, thereby facilitating the development of preventive and therapeutic strategies. Another limitation is the absence of analyses on different stroke subtypes. It is noteworthy that humans are typically exposed to mixtures of multiple PFAS, which may influence cerebral vascular homeostasis through synergistic or antagonistic interactions. Furthermore, factors such as the age at initial exposure, cumulative dose, and non‐monotonic dose‐response relationships, particularly at low doses, may also modulate the impact of PFAS on the cerebrovascular system. While the present study systematically evaluated the association between PFOS exposure and stroke risk using a multi‐faceted approach—including multivariable adjustment, WQS modeling, PLS‐DA, and molecular docking with kinetic simulations—the establishment of causality is constrained by the inherent limitations of its cross‐sectional design. Given that PFAS (e.g., PFOS) possess long biological half‐lives and their serum concentrations reflect long‐term cumulative exposure, a temporal discrepancy exists with stroke, which is an acute clinical event. Consequently, our statistical analyses cannot definitively establish the temporal sequence between exposure and outcome, and the possibility of reverse causality—wherein the stroke event itself might alter PFAS metabolism or distribution—cannot be entirely excluded. Overcoming this limitation necessitates future prospective cohort studies and the integration of genetic instrumental variables identified through large‐scale genome‐wide association studies (GWAS). The application of Mendelian randomization (MR) analysis using these instruments would substantially strengthen causal inference.

Notably, this study revealed a complex, non‐monotonic association between PFNA exposure and stroke risk. Specifically, lower concentrations (Q1, Q2) of PFNA were negatively associated with stroke risk (OR < 1), while higher concentrations (Q4) were positively associated. This observation suggests that the biological effects of PFNA are likely dose‐dependent. We hypothesize that this apparent protective effect at low concentrations could be attributed to hormesis, a phenomenon whereby low‐level exposure to a stressor activates adaptive regulatory pathways (e.g., anti‐inflammatory or antioxidant responses), conferring transient protective effects. However, at higher exposure levels, the deleterious mechanisms of PFNA (such as disruption of the blood‐brain barrier, induction of vascular inflammation, and oxidative damage) likely become predominant, ultimately overwhelming any potential low‐dose benefits and leading to a net adverse outcome (Chang et al. 2024). It is crucial to emphasize that this protective association is an observational finding from a cross‐sectional study, and its underlying biological mechanisms remain speculative. Therefore, it should not be interpreted as evidence of any beneficial effect from PFNA exposure.

In conclusion, this study, to our knowledge, is the first to employ an integrative analytical strategy to identify PFOS as a key PFAS monomer associated with an elevated risk of stroke. By elucidating the mechanistic links to multiple key genes and signaling pathways, these findings help address previous inconsistencies regarding the association between PFAS and stroke. However, these findings rely on computational models and predictive data, lacking direct experimental validation. Moving forward, we plan to further validate the interactions between PFOS and targets such as AKT1 and HSP90AA1 through cellular and animal experiments and assess their role in stroke pathology. Looking ahead, the complex relationship between PFOS exposure and stroke warrants further investigation. The public health implications are substantial, as exposure is linked to the development of stroke risk factors and broader clinical manifestations of cardiovascular disease (CVD). By identifying putative molecular targets and potential mechanisms underlying PFOS‐mediated stroke pathogenesis, our findings provide critical insights that can inform the development of evidence‐based preventive strategies and regulatory policies aimed at mitigating population‐level exposure.

## 5 Conclusion

Employing an integrative methodology that combines data from the NHANES database, network toxicology, and molecular docking analysis, this study elucidates the pathogenic role of PFOS in stroke. Our findings suggest that PFOS exposure promotes atherosclerosis and neurovascular damage through the synergistic disruption of lipid metabolism, inflammation, and oxidative stress, wherein the PI3K/AKT pathway emerges as a central regulatory hub governing neuroinflammation and dysregulated apoptosis. Furthermore, this pathway dynamically interacts with key targets, including AKT1 and HSP90AA1, to exacerbate stroke‐related pathologies. Collectively, this multi‐faceted approach enhances the understanding of how environmental pollutants such as PFAS influence the onset and progression of cerebrovascular diseases, thereby supporting the formulation of multidisciplinary preventive and mitigative strategies.

## Author Contributions

J.Y.J. and Z.S.P. were responsible for the study's design and conception, wrote the initial draft, and revised and integrated contributions from the other authors. J.Y.J., Z.S.P., and W.H.Y. conceptualized the study and handled the direct acquisition and analysis of data. L.M., H.X.Y., F.R., and T.M.J. conducted the statistical analysis of the NHANES database. L.L. and T.E.J. provided guiding suggestions for the modification in molecular dynamics. L.Y.H., J.Z.H., F.Q.W., W.Y.P., L.W.S., and Z.X.Y. were in charge of the network toxicology and molecular docking sections. Z.S.P. and L.Y. oversaw the quality review and finalization of the article. The corresponding author confirms that all listed authors meet the authorship criteria and that no qualifying authors have been omitted. All authors reviewed and approved the final manuscript during the repair process.

## Funding

This study was supported by National Natural Science Foundation of China (81804022).

## Conflicts of Interest

The authors declare no conflicts of interest.

## Peer Review

The peer review history for this article is available at https://publons.com/publon/10.1002/brb3.71014.

## Supporting information




**Supplementary Material**: brb371014‐sup‐0001‐SuppMat.docx

## Data Availability

All data generated or analyzed during this study are included in the published article and its Supplementary Information file. Raw data (Excel spreadsheets) used in the statistical analyses are available upon request from the corresponding author (Yan Lu).
