# Supplementary material for: Exploring the Association Between Per‐ and Polyfluoroalkyl Substances Exposure and the Risk of Stroke: A Systematic Investigation Using NHANES Data Analysis, Network Toxicology and Molecular Docking Approaches
Source: Brain Behav. 2025 Oct 24;15(10):e71014. doi: 10.1002/brb3.71014 (PMC12551669; doi:10.1002/brb3.71014)
Supplement: Supplementary file 1 — Supplementary Material: brb371014‐sup‐0001‐SuppMat.docx [file BRB3-15-e71014-s001.docx]

**Supplementary materials**

**eMethods: WQS Model**

To assess the combined exposure effects of multiple per- and polyfluoroalkyl substances (PFAS), this study developed a weighted quantile sum (WQS) model using R version 4.3.3 and the gWQS package. During model development, PFAS concentrations were first quantile-split based on their distribution, using quartile truncation to mitigate the influence of skewness and outliers. Weighted constraints were then applied to ensure values ranged from 0 to 1 with a sum of 1, yielding an interpretable composite exposure index. Based on prior epidemiological evidence indicating adverse cardiovascular outcomes from PFAS exposure, we hypothesized that all PFAS would exhibit a consistent “adverse direction” of influence on stroke risk, implying the WQS index would be positively correlated with stroke risk. Therefore, a positive weight constraint was applied to all PFAS components in the model (i.e., parameter b1_pos = TRUE). To ensure reproducibility, a fixed random seed (seed = 1234) was set. The dataset was randomly split into a training set (40%) and a validation set (60%) at a 4:6 ratio. The training set was used to estimate weights for each PFAS via an adaptive resampling algorithm (Bootstrap runs = 1000), with the sum of weights constrained to 1 and disabling zero weight compression. The final number of PFAS compounds (p) included in the model was 10. Therefore, we employed a threshold of ≥ 1/10 = 0.1 to identify the pollutants with the most significant contributions to mixed exposure. The validation set was used to fit the final association model between the WQS composite index and stroke risk, estimating the odds ratio (OR) and its 95% confidence interval through 1000 Bootstrap calculations.

**eTable 1. Website URLs for database and tools used in present study.**

| **No. Web tool or software** | **Uniform resource locator** |
| --- | --- |
| admetSAR 3.0 | http://lmmd.ecust.edu.cn/admetsar3/ |
| AutoDock Vina_v1.5.6 | https://vina.scripps.edu/ |
| ChEMBL database | https://www.ebi.ac.uk/chembl/ |
| CTD database | https://ctdbase.org/ |
| Cytoscape_v3.10.3 | https://cytoscape.org/ |
| Cytoscape Hubba | https://apps.cytoscape.org/download/stats/cytohubba/ |
| Cytoscape MCODE | https://apps.cytoscape.org/apps/mcode |
| DisGeNET database | https://disgenet.com/ |
| GeneCards database | https://www.genecards.org/ |
| NHANES | https://www.cdc.gov/nchs/nhanes/index.htm |
| NHANES Laboratory Data Overview | https://www.cdc.gov |
| PharmMapper database | http://www.lilab-ecust.cn/pharmmapper/ |
| ProTox 3.0 | https://tox.charite.de |
| PubChem database | https://pubchem.ncbi.nlm.nih.gov/ |
| Pymol 3.1 | https://www.pymol.org/ |
| RCSB PDB | <https://www.rcsb.org> |
| STRING tool | https://cn.string-db.org/ |
| TargetNet database | http://targetnet.scbdd.com |
| Uniprot database | https://www.uniprot.org/ |

**eTable 2. Baseline characteristics of participants (n = 8,081), NHANES 2003–2012.**

| **Type** | **Classification** | **None-Stroke (N = 7,775)** | **Stroke (N = 306)** | **total (N = 8,081)** | **P-value** |
| --- | --- | --- | --- | --- | --- |
| Ethnicity, n (%) | Mexican American | 1,339 (17.2%) | 35 (11.4%) | 1,374 (17%) | <0.001* |
|  | Non-Hispanic White | 621 (8%) | 10 (3.3%) | 631 (7.8%) |  |
|  | Non-Hispanic Black | 3,696 (47.5%) | 165 (53.9%) | 3,861 (47.8%) |  |
|  | Other Hispanic | 1,576 (20.3%) | 79 (25.8%) | 1,655 (20.5%) |  |
|  | Other Race | 543 (7%) | 17 (5.6%) | 560 (6.9%) |  |
| Gender, n (%) | Female | 3,987 (51.3%) | 152 (49.7%) | 4,139 (51.2%) | 0.311 |
|  | Male | 3,788 (48.7%) | 154 (50.3%) | 3,942 (48.8%) |  |
| BMI, n (%) | <25 | 2,282 (29.4%) | 69 (22.5%) | 2,351 (29.1%) | 0.037* |
|  | 25-29.9 | 2,727 (35.1%) | 118 (38.6%) | 2,845 (35.2%) |  |
|  | ≥30 | 2,766 (35.6%) | 119 (38.9%) | 2,885 (35.7%) |  |
| Hypertension, n (%) | No | 5,226 (67.2%) | 69 (22.5%) | 5,295 (65.5%) | <0.001* |
|  | Yes | 2,549 (32.8%) | 237 (77.5%) | 2,786 (34.5%) |  |
| Diabetes, n (%) | No | 6,942 (89.3%) | 214 (69.9%) | 7,156 (88.6%) | <0.001* |
|  | Yes | 833 (10.7%) | 92 (30.1%) | 925 (11.4%) |  |
| CHD, n (%) | No | 7,487 (96.3%) | 258 (84.3%) | 7,745 (95.8%) | <0.001* |
|  | Yes | 288 (3.7%) | 48 (15.7%) | 336 (4.2%) |  |
| Smoking, n (%) | Every day | 1,328 (17.1%) | 62 (20.3%) | 1,390 (17.2%) | 0.181 |
|  | Sometimes | 286 (3.7%) | 7 (2.3%) | 293 (3.6%) |  |
|  | Never | 6,161 (79.2%) | 237 (77.5%) | 6,398 (79.2%) |  |
| Alcohol, n (%) | Yes | 2716 (34.9%) | 125 (40.8%) | 2,841 (35.2%) | 0.039* |
|  | No | 5,059 (65.1%) | 181 (59.2%) | 5,240 (64.8%) |  |
| Education, n (%) | High School or less | 3,928 (50.5%) | 197 (64.4%) | 4,125 (51%) | <0.001* |
|  | Some College or AA degree | 2,195 (28.2%) | 73 (23.9%) | 2,268 (28.1%) |  |
|  | College graduate or above | 1,652 (21.2%) | 36 (11.8%) | 1,688 (20.9%) |  |
| PIR, n (%) | ≤1 | 61 (19.9%) | 1,425 (18.3%) | 1,486 (18.4%) | <0.001* |
|  | 1-3 | 176 (57.5%) | 3,659 (47.1%) | 3,835 (47.5%) |  |
|  | >3 | 69 (22.5%) | 2,691 (34.6%) | 2,760 (34.2%) |  |
| PA, n (%) | Never | 4,152 (53.4%) | 210 (68.6%) | 4,362 (54%) | 0.008* |
|  | Vigorous | 1,644 (21.1%) | 31 (10.1%) | 1,675 (20.7%) |  |
|  | Moderate | 1,979 (25.5%) | 65 (21.2%) | 2,044 (25.3%) |  |
| Age | Mean ± SD | 49.0 ± 18.2 | 67.8 ± 13.2 | 49.7 ± 18.4 | <0.001* |
| BMI | Mean ± SD | 28.8 ± 6.7 | 29.5 ± 6.5 | 28.8 ± 6.7 | 0.106 |

Continuous measures are presented as mean ± standard deviation (SD). Categorical variables are presented as weighted percentages. Abbreviations: BMI, Body Mass Index; CHD, Coronary heart disease; PA, Physical activity; PIR, Poverty Income Ratio. * *p* < 0.05.

**eTable 3. Logistic regression analysis of the association between the blood serum PFAS levels and stroke risk in the participants, weighted.**

| **PFAS types** | **Quarter** | **None-Stroke (N = 7,775)** | **Stroke (N = 306)** | **Model 1** | **Model 2** | **Model 3** |
| --- | --- | --- | --- | --- | --- | --- |
| Perfluorooctanoic acid (PFOA) | Q1 | 1,891 (24.3%) | 57 (18.6%) | Reference | Reference | Reference |
|  | Q2 | 1,952 (25.1%) | 74 (24.2%) | 1.26 (0.89-1.79, p = 0.201) | 1.22 (0.85-1.74, p = 0.287) | 1.04 (0.72-1.50, p = 0.826) |
|  | Q3 | 1,950 (25.1%) | 78 (25.5%) | 1.33 (0.94-1.88, p = 0.110) | 1.26 (0.88-1.80, p = 0.200) | 1.15 (0.88-1.80, p = 0.200) |
|  | Q4 | 1,982 (25.5%) | 97 (31.7%) | 1.62 (1.16-2.27, p = 0.004) * | 1.44 (1.02-2.02, p = 0.039) * | 1.28 (0.90-1.82, p = 0.175) |
| Perfluorooctane sulfonic acid (PFOS) | Q1 | 1,974 (25.4%) | 45 (14.7%) | Reference | Reference | Reference |
|  | Q2 | 1,935 (24.9%) | 60 (19.6%) | 1.36 (0.92-2.01, p = 0.124) | 1.23 (0.82-1.83, p = 0.313) | 1.13 (0.75-1.69, p = 0.564) |
|  | Q3 | 1,937 (24.9%) | 79 (25.8%) | 1.79 (1.23-2.59, p = 0.002) * | 1.45 (0.99-2.12, p = 0.056) | 1.25 (0.85-1.85, p = 0.258) |
|  | Q4 | 1,929 (24.8%) | 122 (39.9%) | 2.77 (1.96-3.93, p < 0.001) * | 2.03 (1.42-2.90, p < 0.001) * | 1.59 (1.09-2.31, p = 0.016) * |
| Perfluorohexane sulfonic acid (PFHXS) | Q1 | 1,819 (23.4%) | 54 (17.6%) | Reference | Reference | Reference |
|  | Q2 | 1,931 (24.8%) | 75 (24.5%) | 1.31 (0.92-1.87, p = 0.139) | 1.21 (0.84-1.73, p = 0.315) | 1.00 (0.69-1.45, p = 0.996) |
|  | Q3 | 2,022 (26%) | 78 (25.5%) | 1.30 (0.91-1.85, p = 0.145) | 1.09 (0.76-1.56, p = 0.646) | 0.83 (0.57-1.22, p = 0.350) |
|  | Q4 | 2,003 (25.8%) | 99 (32.4%) | 1.66 (1.19-2.33, p = 0.003) * | 1.39 (0.98-1.97, p = 0.062) | 1.08 (0.75-1.56, p = 0.664) |
| 2-(N-Ethyl-perfluorooctane sulfonamido) acetic acid (N-ETFOSAA) | Q1 | 3,227 (41.5%) | 98 (32%) | Reference | Reference | Reference |
|  | Q2 | 4,548 (58.5%) | 208 (68%) | 1.51 (1.18-1.92, p = 0.001) * | 1.51 (1.17-1.93, p = 0.001) * | 1.53 (1.18-1.97, p = 0.001) * |
| 2-(N-Methyl-perfluorooctane sulfonamido) acetic acid (N-MEFOSAA) | Q1 | 1,542 (19.8%) | 28 (9.2%) | Reference | Reference | Reference |
|  | Q2 | 1,748 (22.5%) | 69 (22.5%) | 2.17 (1.39-3.39, p < 0.001) * | 2.23 (1.42-3.50, p = 0.001) * | 2.19 (1.38-3.46, p = 0.001) * |
|  | Q3 | 2,271 (29.2%) | 91 (29.7%) | 2.21 (1.44-3.39, p < 0.001) * | 2.11 (1.37-3.26, p = 0.001) * | 1.94 (1.25-3.01, p = 0.003) * |
|  | Q4 | 2,214 (28.5%) | 118 (38.6%) | 2.94 (1.93-4.45, p < 0.001) * | 2.37 (1.55-3.63, p < 0.001) * | 2.00 (1.30-3.08, p = 0.002) * |
| Perfluoroheptanoic acid (PFHPA) | Q1 | 3,082 (39.6%) | 91 (29.7%) | Reference | Reference | Reference |
|  | Q2 | 1,467 (18.9%) | 73 (23.9%) | 1.69 (1.23-2.31, p = 0.001) * | 1.54 (1.11-2.12, p = 0.009) * | 1.50 (1.08-2.08, p = 0.016) * |
|  | Q3 | 3,226 (41.5%) | 142 (46.4%) | 1.49 (1.14-1.95, p = 0.004) * | 1.51 (1.15-1.98, p = 0.003) * | 1.55 (1.17-2.05, p = 0.002) * |
| Perfluorononanoic acid (PFNA) | Q1 | 1,937 (24.9%) | 79 (25.8%) | Reference | Reference | Reference |
|  | Q2 | 1,810 (23.3%) | 49 (16%) | 0.66 (0.46-0.95, p = 0.026) * | 0.63 (0.44-0.91, p = 0.015) * | 0.63 (0.43-0.91, p = 0.014) * |
|  | Q3 | 2,077 (26.7%) | 77 (25.2%) | 0.91 (0.66-1.25, p = 0.559) | 0.89 (0.44-1.24, p = 0.490) | 0.85 (0.61-1.19, p = 0.334) |
|  | Q4 | 1,951 (25.1%) | 101 (33%) | 1.27 (0.94-1.72, p = 0.120) | 1.12 (0.82-1.52, p = 0.487) | 1.03 (0.75-1.42, p = 0.0857) |
| Perfluoroundecanoic acid (PFUA) | Q1 | 1,541 (19.8%) | 37 (12.1%) | Reference | Reference | Reference |
|  | Q2 | 2,231 (28.7%) | 94 (30.7%) | 1.75 (1.19-2.58, p = 0.004) * | 1.86 (1.25-2.75, p = 0.002) * | 1.87 (1.26-2.79, p = 0.002) * |
|  | Q3 | 2,065 (26.6%) | 90 (29.4%) | 1.82 (1.23-2.68, p = 0.003) * | 1.80 (1.21-2.68, p = 0.004) * | 1.67 (1.12-2.50, p = 0.012) * |
|  | Q4 | 1,938 (24.9%) | 85 (27.8%) | 1.83 (1.23-2.70, p = 0.003) * | 1.80 (1.21-2.69, p = 0.004) * | 1.57 (1.04-2.38, p = 0.031) * |
| Pefluorodecanoic acid (PFDA) | Q1 | 1,772 (22.8%) | 60 (19.6%) | Reference | Reference | Reference |
|  | Q2 | 2,117 (27.2%) | 88 (28.8%) | 1.23 (0.88-1.71, p = 0.229) | 1.29 (0.92-1.82, p = 0.140) | 1.24 (0.87-1.75, p = 0.230) |
|  | Q3 | 1,289 (16.6%) | 41 (13.4%) | 0.94 (0.63-1.41, p = 0.761) | 1.05 (0.69-1.58, p = 0.830) | 1.06 (0.70-1.61, p = 0.776) |
|  | Q4 | 2,597 (33.4%) | 117 (38.2%) | 1.33 (0.97-1.83, p = 0.077) | 1.37 (0.99-1.89, p = 0.058) | 1.26 (0.90-1.76, p = 0.175) |
| Perflurododecanoic acid (PFDO) | Q1 | 3,188 (41%) | 96 (31.4%) | Reference | Reference | Reference |
|  | Q2 | 4,587 (59%) | 210 (68.6%) | 1.52 (1.19-1.94, p < 0.001) * | 1.24 (1.10-1.41, p = 0.001) * | 1.57 (1.22-2.03, p = 0.001) * |

Model 1 has not undergone any adjustments.

Model 2 adjusted for coronary heart disease, hypertension and diabetes.

Model 3 further adjusted age, alcohol, BMI, education, ethnicity, gender, smoking, physical activity and poverty income ratio on the basis of Model 2.

* *p* < 0.05.

**eTable 4. PROTox -Prediction of toxicity of chemicals.**

| **Classification** | **Target** | **Prediction** | **Probability** |
| --- | --- | --- | --- |
| Organ toxicity | Hepatotoxicity | Inactive | 0.89 |
| Organ toxicity | Neurotoxicity | Inactive | 0.94 |
| Organ toxicity | Nephrotoxicity | Inactive | 0.52 |
| Organ toxicity | **Respiratory toxicity** | **Active** | **0.54** |
| Organ toxicity | Cardiotoxicity | Inactive | 0.88 |
| Toxicity end points | Carcinogenicity | Inactive | 0.68 |
| Toxicity end points | Immunotoxicity | Inactive | 0.99 |
| Toxicity end points | Mutagenicity | Inactive | 0.64 |
| Toxicity end points | Cytotoxicity | Inactive | 0.75 |
| Toxicity end points | **BBB-barrier** | **Active** | **0.91** |
| Toxicity end points | Ecotoxicity | Inactive | 0.61 |
| Toxicity end points | Clinical toxicity | Inactive | 0.76 |
| Toxicity end points | Nutritional toxicity | Inactive | 0.86 |

**eTable 5. TOXICOPHORE RULES.**

| **Classification** | **Probability** | **labels** |
| --- | --- | --- |
| Neurotoxicity | 100% | ● |
| DILI | 47.8% | ● |
| hERG 1uM | 3.9% | ● |
| hERG 10uM | 29.3% | ● |
| hERG 30uM | 40.69% | ● |
| hERG 1-10uM | 3.4% | ● |
| hERG 10-30uM | 33% | ● |
| Respiratory toxicity | 27% | ● |
| Nephrotoxicity | 60.6% | ● |
| Eye corrosion | 90.2% | ● |
| Eye irritation | 95.8% | ● |
| Skin corrosion | 78% | ● |
| Skin irritation | 59.6% | ● |
| Skin sensitisation | 53.7% | ● |
| Acute dermal toxicity | 72.4% | ● |

The corresponding relationships of the three labels (green, yellow, red) are as follows: excellent, medium and poor.

**eTable 6. Potential targets of PFOS induced stroke (183).**

| **Potential targets of PFOS induced stroke** | | | | | | |
| --- | --- | --- | --- | --- | --- | --- |
| RARA | CYP1A1 | GSTP1 | WAS | SHMT1 | SIRT1 | ABCG2 |
| NR1H3 | CYP3A5 | BMP2 | MAPK10 | REN | DNMT1 | CNR1 |
| UGT1A1 | CYP1A2 | MAOB | NOS3 | SOD2 | CYP17A1 | HDAC6 |
| SCD | CYP2C9 | ESR1 | GSR | SULT1E1 | RELA | NOS1 |
| FABP4 | CYP2C19 | MAPK14 | PDE4D | PPARA | PLG | CDK5 |
| EIF4EBP1 | CYP2C8 | TTR | HSPA8 | NR3C1 | TUBA1A | CACNA1H |
| ALOX5AP | CYP2D6 | CYP19A1 | MAPK8 | PCK1 | PTGS1 | CXCR4 |
| SLC7A11 | CYP2B6 | PGR | CDK2 | EPHX2 | RAC1 | CTSL |
| SLC1A2 | CYP1B1 | MMP13 | TGFBR1 | PARP1 | PLAT | ELANE |
| CYP2J2 | CYP2E1 | CASP3 | IGF1R | FGFR1 | MMP9 | TUBB2B |
| AR | CYP2R1 | LCN2 | PTPN11 | NQO1 | TNF | SLC6A9 |
| FABP3 | SLC7A5 | APOA2 | F10 | NR1I2 | BRAF | ESR2 |
| FGF1 | ACSL4 | THRB | HSD11B1 | XDH | CDC42 | PNP |
| FASN | HDAC1 | CASP7 | CFD | MAOA | SERPINE1 | SELE |
| FABP1 | KLRK1 | EGFR | CBS | AHR | SLC6A4 | DRD2 |
| CYP26B1 | SLC1A3 | F2 | MDM2 | CA12 | CASP6 | CASP1 |
| CD36 | BCL6 | AKR1C3 | MMP3 | PTGS2 | MCL1 | FLT1 |
| NR4A2 | KCNK3 | ALB | KDR | APP | PDE3A | PDGFRA |
| SLC23A2 | SLC7A8 | EPHB4 | GSK3B | ADRA2B | ABCB1 | TEK |
| VDR | AOX1 | SRC | HMGCR | MGLL | ACHE | CYP11B2 |
| BIRC2 | ALAD | KIF11 | IGF1 | SCN5A | PTGES | AKT1 |
| GSTO1 | ORM1 | NR3C2 | AKT2 | ALPL | GRIN2B |  |
| GBA1 | GAA | CCNA2 | PGF | HSD11B2 | MAPK11 |  |
| NCOR2 | ERBB2 | MET | HSP90AA1 | NOS2 | KCNH2 |  |
| PARK7 | SLC10A1 | ANXA5 | CDK6 | NR2F2 | TERT |  |
| PPARG | RPS6 | GSTA1 | CTSB | PTK2 | RPS6KB1 |  |
| CYP3A4 | MAPK1 | PDPK1 | MMP12 | CASP9 | FDFT1 |  |

**eFigure 1. Flowchart of Study Participants in NHANES.**


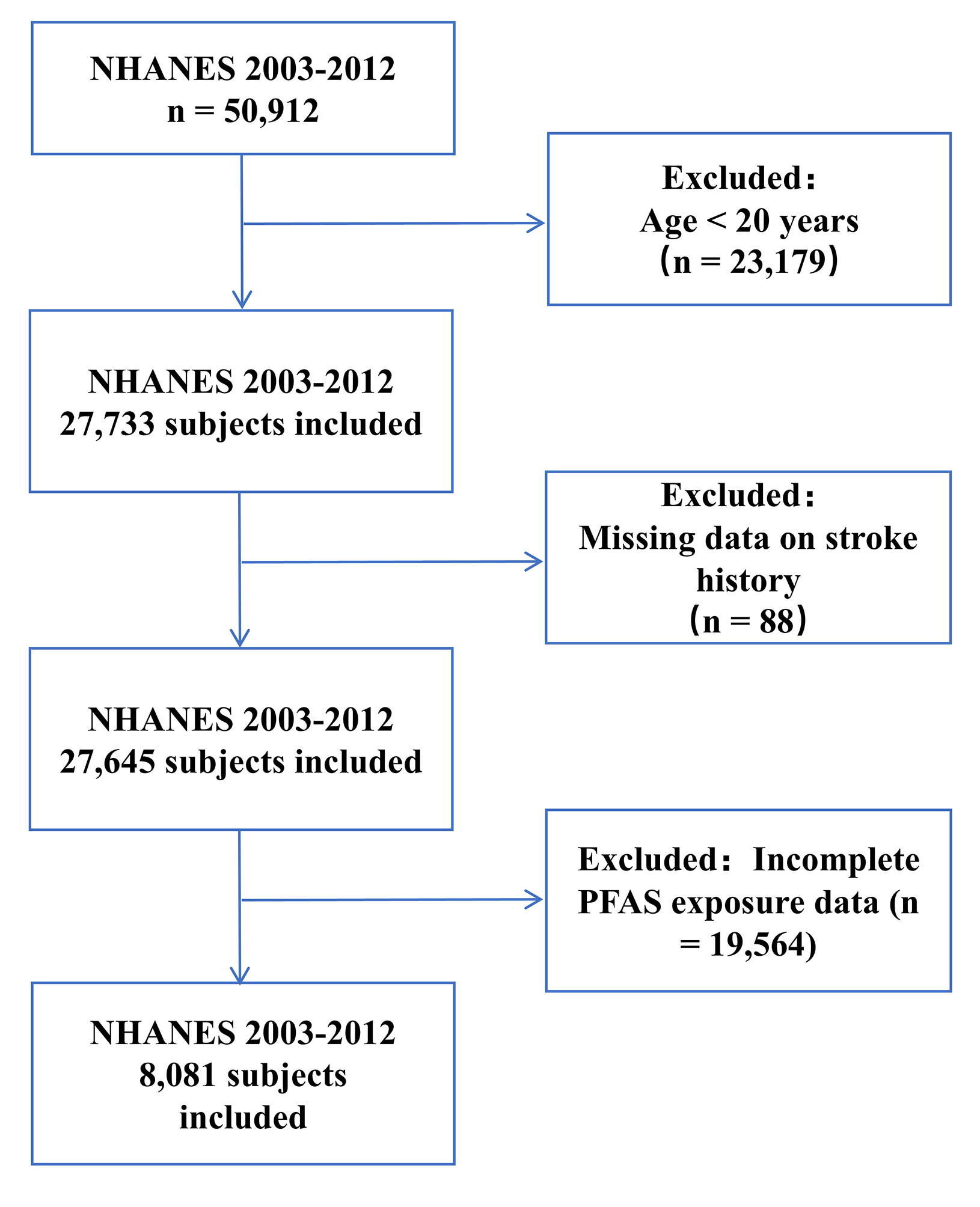


**eFigure 2. Association Between Serum PFOS Levels and Stroke Risk.**


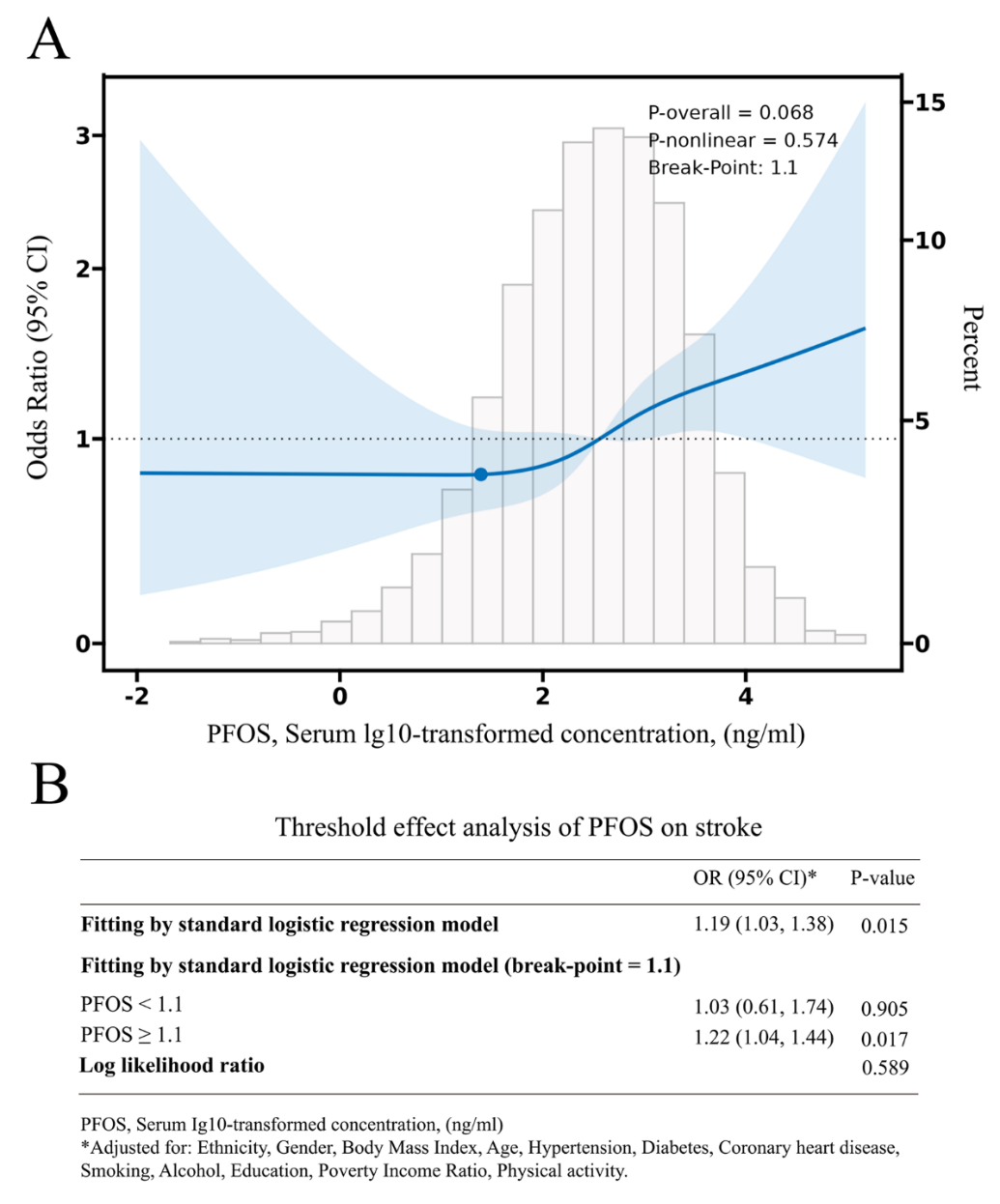


**A**: Adjusted smooth curve showing the association between log-transformed serum PFOS levels and the probability of stroke.

X-Axis (PFOS, Serum lg10-transformed concentration, ng/ml): The x-axis represents the serum concentration of PFOS, which is transformed by the base-10 logarithm. Y-Axis (Odds Ratio (95% CI)): The y-axis represents the odds ratio (OR) with its 95% confidence interval (CI). Break Point: The "Break-Point" at 1.1 represents a threshold where the relationship between PFOS concentration and stroke risk may change. P-overall: This p-value refers to the overall statistical significance of the relationship between PFOS concentration and stroke risk. P-nonlinear: This p-value assesses whether the dose-response curve is nonlinear. Histograms (Gray Bars): The histograms in the background show the distribution of PFOS serum concentrations among the study population. Blue Line: The blue line represents the estimated odds ratio from the RCS model, illustrating the relationship between PFOS concentration and stroke risk. The confidence intervals are shaded in blue. Percent (Right Y-Axis): The secondary y-axis on the right-hand side represents the percentage of individuals in the study sample with a given PFOS serum concentration.

**B**: Threshold efect analysis of PFOS on stroke.

**eFigure 3.** Subgroup analysis for the relationship between PFOS and the risk of stroke.


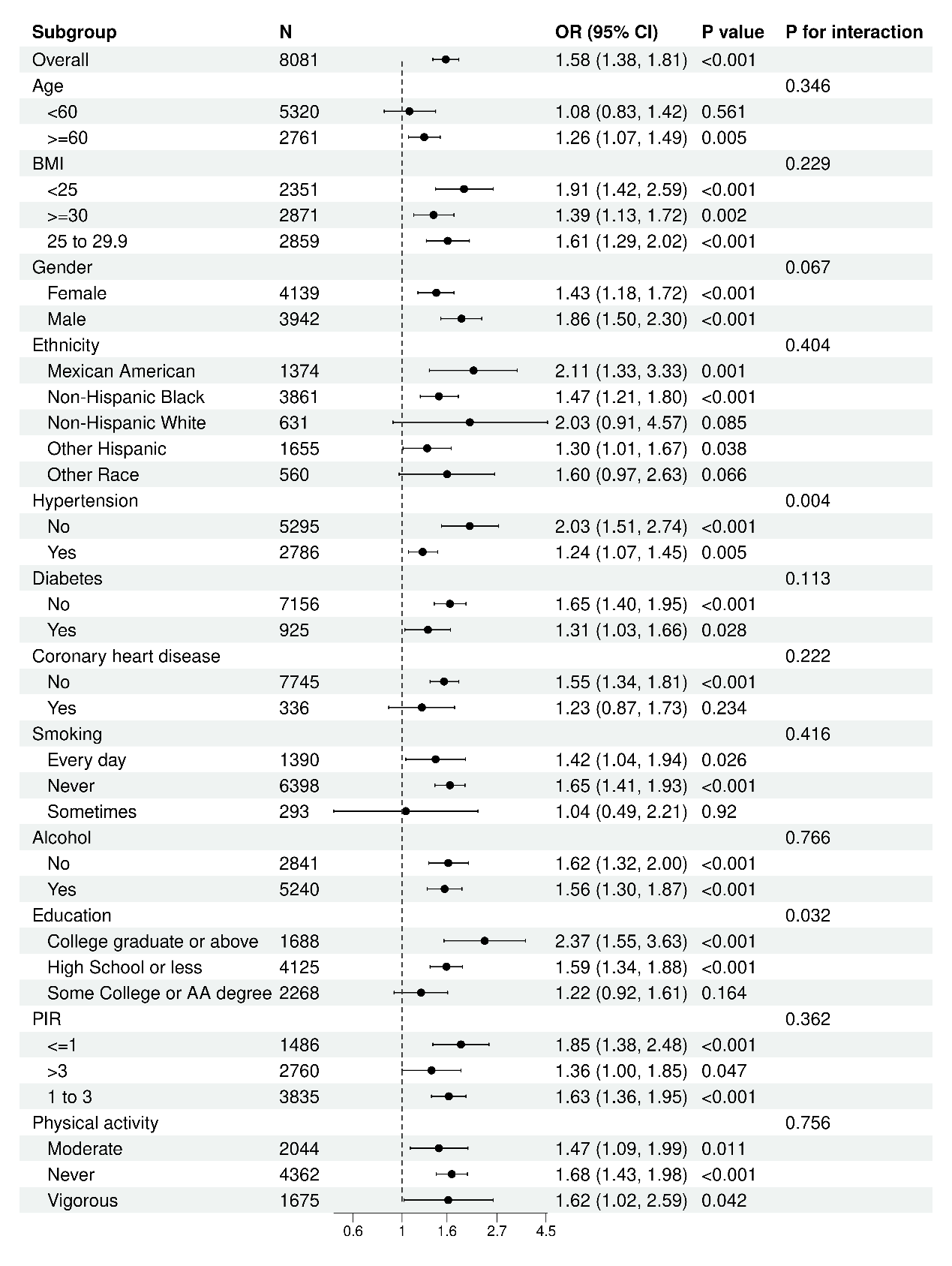


The figure shows the ratio of ratios (OR) with 95% confidence intervals (CIs) for different subgroups assessing the effect of PFOS on stroke risk. The p-value for the interaction is indicated in the last column. Significant correlations (p < 0.05) are indicated by circles for each subgroup. Statistical significance is indicated by p-values < 0.05. All models are adjusted for relevant confounders.

**eFigure 4.** This network highlights functional associations between PFOS and targets related to stroke.


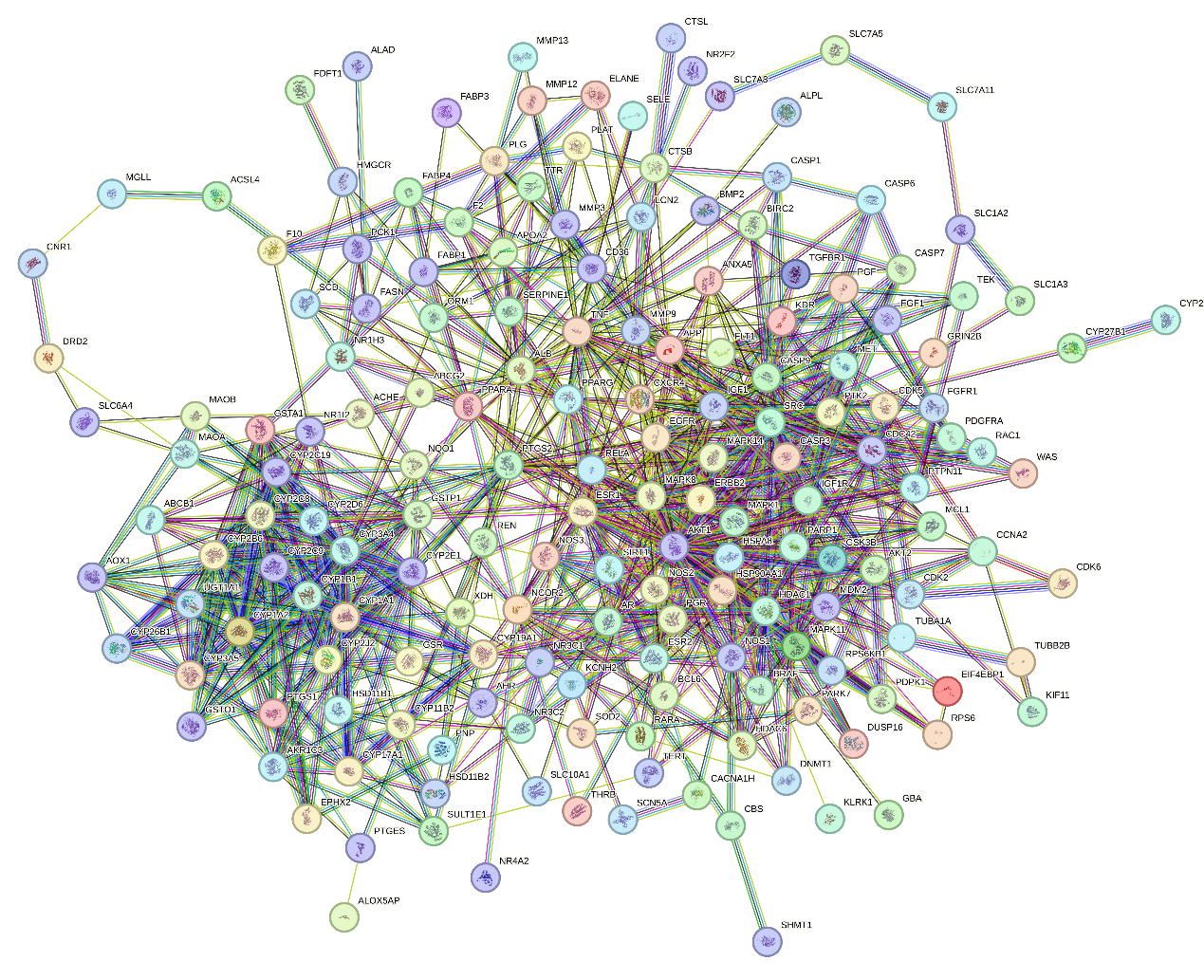


The PPI network was constructed using the STRING database with a confidence score threshold of ≥ 0.9. Nodes represent proteins, and edges indicate interactions between them.

**eFigure 5.** The detailed classification display of hub genes enriched in the KEGG signaling pathways.
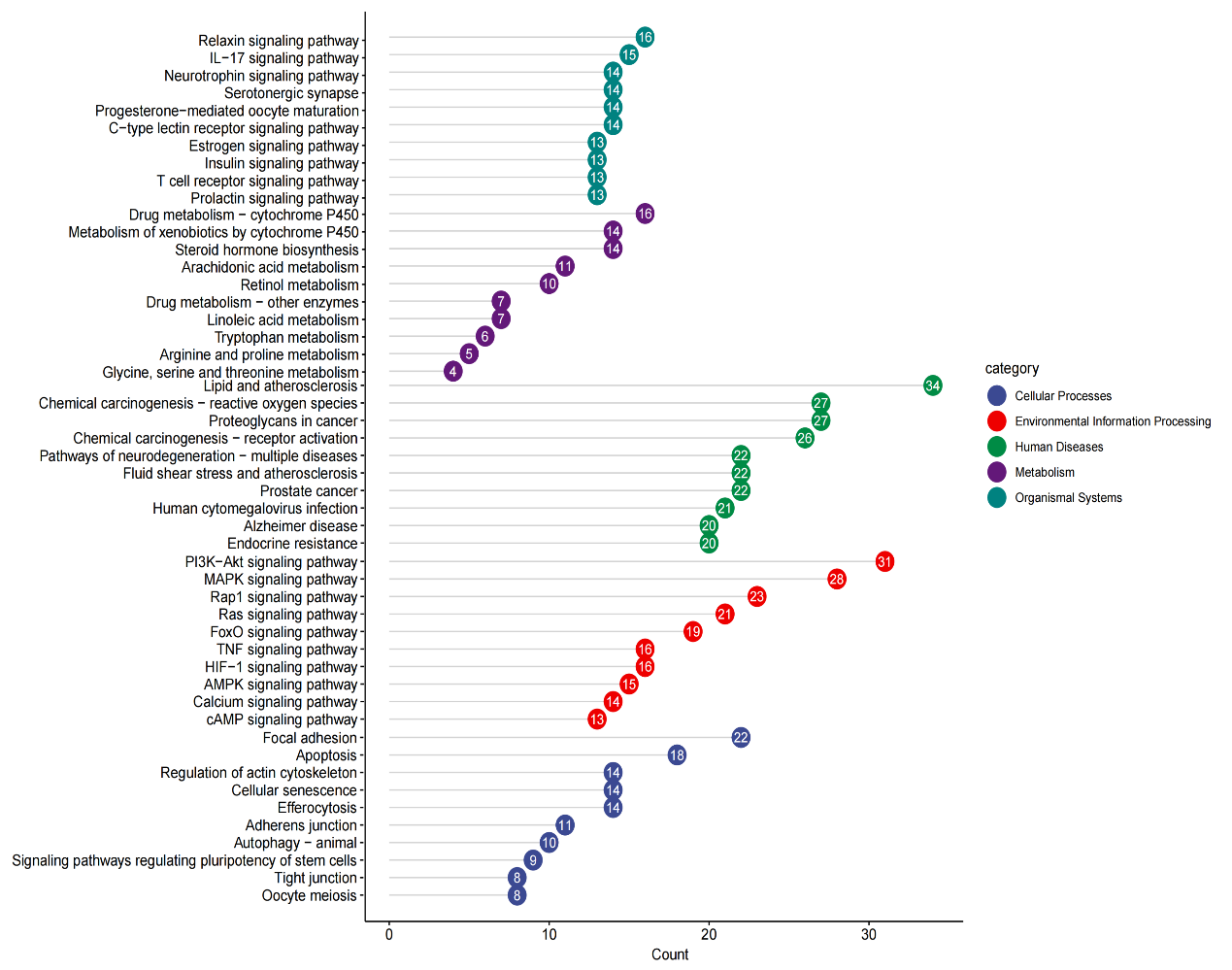


This figure shows the classification and count of hub genes enriched in various KEGG signaling pathways.

Cellular Processes (blue): Pathways involved in basic cellular functions and processes.

Environmental Information Processing (red): Pathways related to the response to external signals and environmental stressors.

Human Diseases (green): Pathways associated with various diseases and health conditions.

Metabolism (purple): Pathways involved in metabolic processes and energy regulation.

Organismal Systems (teal): Pathways related to organismal systems such as immune and endocrine systems.
